# New Insights into the Molecular Evolution of *Tapirus pinchaque* (Tapiridae, Perissodactyla) and the Rise and Fall of *Tapirus kabomani* as a Full Species

**DOI:** 10.3390/genes15121537

**Published:** 2024-11-28

**Authors:** Manuel Ruiz-García, Armando Castellanos, Franz Kaston, Myreya Pinedo-Castro, Joseph Mark Shostell

**Affiliations:** 1 Laboratorio de Genética de Poblaciones-Biología Evolutiva, Unidad de Genética, Departamento de Biología, Facultad de Ciencias, Pontificia Universidad Javeriana, Cra 7A No 43-82, Bogotá 110311, Colombia; pinedom@javeriana.edu.co; 2 Instituto Nacional de Biodiversidad (INABIO), Pje Rumipamba N.341 y Av. De los Shyris, Quito 170135, Ecuador; iznachi@gmail.com; 3 Andean Bear Fundation, La Isla, Quito 170521, Ecuador; 4 Fundación Nativa, Apartado Aéreo 59199, Bogotá 110121, Colombia; tapirlanudo@hotmail.com; 5 Department of Math Science and Technology, University of Minnesota Crookston, Crookston, MN 56716, USA; joseph.shostell@gmail.com

**Keywords:** *BRCA1*, demographic changes, gene diversity, *IRBP*, mitogenomes and nuclear gene sequences, phylogenetic inferences, *RAG 1-2*, South America, spatial structure, *Tapirus kabomani*, *Tapirus pinchaque*

## Abstract

Large wild mammals are extremely important in their respective ecological communities and are frequently considered to be emblematic. This is the case of the different tapir species, the largest terrestrial mammals from the Neotropics. Despite their large size and being objects of interest for many naturalists, the field still lacks critical genetics and systematics information about tapir species. In the current work, we analyzed four molecular datasets (mitogenomes, and three nuclear genes, *RAG 1-2*, *IRBP*, and *BRCA1*) of two South American tapirs: the Andean tapir (*Tapirus pinchaque*) and the alleged new species of tapir, *Tapirus kabomani*. We derived four main findings. (1) Our molecular phylogenetic analyses showed *T. pinchaque* as the youngest tapir branch in Neotropics and a sister species of *Tapirus terrestris*. This contradicts the traditional morphological observations of renowned zoologists and paleontologists, who considered *T. pinchaque* as the oldest Neotropical tapir. (2) Our data does not support that the alleged *T. kabomani* is a full species. Rather, it is a specific group within *T. terrestris*. (3) *T. pinchaque* is the Neotropical tapir species which yielded the lowest levels of genetic diversity (both for mitochondrial and nuclear data). (4) The spatial genetic structure for *T. pinchaque* shows differences depending on the type of molecular marker used. With mitogenomes, the spatial structure is relatively weak, whereas with two nuclear genes (*RAG 1-2* and *IRBP*), the spatial structure is highly significant. Curiously, for the other nuclear gene (*BRCA1*), the spatial structure is practically nonexistent. In any case, the northernmost population of *T. pinchaque* we studied (Los Nevados National Park in Colombia) was in a peripatric situation and was the most genetically differentiated. This is important for the adequate conservation of this population. (5) *T. pinchaque* showed clear evidence of population expansion during the last part of the Pleistocene, a period during which the dryness and glacial cold extinguished many large mammals in the Americas. However, *T. pinchaque* survived and spread throughout the Northern Andes.

## 1. Introduction

Large mammals are often considered umbrella or emblematic species whose presence may greatly affect community structure [1]. In general, the extraction of large vertebrates, especially those at the top of the trophic chain, can provoke strong extinction waves in complex natural systems [2]. These species can be considered as key to overall community structure because they live sympatrically with many other species. In Latin America, three tapir species have been traditionally accepted, the Baird or Central American tapir (*Tapirus bairdii*), the lowland or Brazilian tapir (*Tapirus terrestris*), and the mountain tapir (*Tapirus pinchaque*) [3]. A decade ago, it was claimed that a fourth tapir species, *Tapirus kabomani*, existed on the Amazon [4,5]. However, authors quickly responded with evidence that *T. kabomani* was probably not a full species, but rather, it was a differentiated form within *T. terrestris* [6,7,8,9]. In fact, in the current paper, we present the strongest evidence to date that *T. kabomani* is a determined clade within *T. terrestris* and not a full species.

One of these tapir species (*T. pinchaque*) lives in the South American Andean mountains. *T. pinchaque* (type locality: Páramo de Sumapaz in the Cundinamarca Department, Colombia; two specimens were taken on the paramos of Quindío and Sumapaz, Colombia by Roulin in 1829, and later the finding was reported [10]) is the smallest of all the living tapir species—having an average shoulder height of 0.90 m, a body length of 1.8 m, and weight of 150 kg. This species is found in humid and cold habitats of 1700–4800 m above sea level (masl) [11] in different types of Andean forests and páramos (open grass lands) in Colombia, Ecuador, and in northern Peru. It is an extremely efficient seed disperser. It was documented that more than 50 Andean plant species depend on tapir feces for germination within Sangay National Park (Ecuador) [11]. Mountain tapir survival is considered a crucial factor for the conservation of the northern Andean wilderness and watershed. It was estimated to have a maximum population of 5000 to 5700 mountain tapirs, based on existing suitable habitat and estimated densities for this tapir species [12]. However, the same authors considered that the population censuses were smaller than 5000 animals, and that the overall mountain tapir population could be closer to 2650–2850 individuals. Currently, seven [13] or eight [12] protected areas contain mountain tapirs in Colombia (Central and Eastern Andean Cordilleras) and six protected areas contain mountain tapirs in Ecuador. In northern Peru, *T. pinchaque* is found only in the provinces of Ayabaca and Huancabamba from the Piura Department, and in Jaen Province from the Cajamarca Department [14], and along the Peru–Ecuador border in the Cordillera del Condor [15]. In the Peruvian area, this species lives in a unique protected area, Tabaconas-Nambaye National Sanctuary. This tapir species is currently classified as an endangered species, as one of the rarest large mammals in the world. Furthermore, this species has been categorized as critically endangered in the Andean area of Colombia, Ecuador, and Peru [16,17].

Scholars have debated about the role of *T. pinchaque* in the origin of tapirs in South America. Hershkovitz [18] analyzed morphological characters and concluded that the ancestor of *T. pinchaque* was the first tapir form to enter South America prior to the formation of the Panamanian Isthmus around 3–4 million years ago (mya) and that the ancestors of *T. terrestris* and *T. bairdii* also independently evolved in North America and they later entered in South America. Thus, the ancestors of these three tapir species (currently present in South America) first appeared in North America at a time when the Isthmus of Panama had not yet been formed. Hershkovitz [19] also concluded that *T. bairdii* was the most recent of these three species. This author distinguished two subspecies of *T. pinchaque*: *T. p. pinchaque* (Roulin) and *T. p. leucogenys* (Gray) with type locality in Páramo de Azuay in the Eastern Cordillera of Ecuador. Haffer [20] showed a different point of view on the origin of the tapirs of South America considering morphological and biogeographical considerations. He considered the ancestor of *T. pinchaque* to be the first form which colonized the Northern Andes during the last Andes rising within the Pliocene. During the Pleistocene, the ancestor of *T. pinchaque* gave rise in the eastern lowlands to the ancestor of *T. terrestris* and in the western lowlands (Choco refugium) to the ancestor of *T. bairdii*, which later migrated to Central America (one unique lineage hypothesis). Another alternative hypothesis suggests that the ancestor of *T. bairdii* originated in a Central American refugium and then migrated towards the Pacific area of what would eventually become the western section of current Colombia [20]. Therefore, the authors concluded that *T. bairdii* was the most recent of the three current tapir lineages in South America [19,20]. Previously, Hatcher [21] concluded that *T. bairdii* was the most specialized tapir and probably derived from *T. pinchaque* through *T. terrestris*. Nevertheless, Simpson [22] noted that *T. pinchaque* was closely related to *T. terrestris*.

A long time later, molecular analysis [7,8,9,23,24,25,26] showed that the ancestor of *T. bairdii* was older than the ancestors of the sister clade *T. pinchaque–T. terrestris* and that the monophyly of the Neotropical tapir species was clear.

Ruiz-García and collaborators [8] provided the first and unique molecular genetic analysis of *T. pinchaque* to date. These authors analyzed 15 mitochondrial genes in 45 specimens (83.1% of the total mitochondrial DNA’s length) and showed the five following noteworthy results: 1. The genetic diversity level of *T. pinchaque* showed 21 different haplotypes with H_d_ = 0.904 ± 0.003 (high genetic diversity) and π = 0.0041 ± 0.0003 (low-medium genetic diversity). The genetic diversity levels for the Colombian and the Ecuadorian mountain tapir populations were similar, although the most unbiased gene diversity statistics were somewhat higher for the Colombian specimens. 2. Genetic heterogeneity tests revealed a low genetic differentiation level between the Colombian and Ecuadorian populations of *T. pinchaque* (G_ST_ = 0.0231, and F_ST_ = 0.056; *p* = 0.714–0.367, no significant). An AMOVA revealed that the percentage of variation between groups (Colombia and Ecuador) was very small (2.27%) and not significant. The percentage of variation among the populations within the groups was also small (6.76%) and not significant. The major percentage of genetic variance within populations was 90.97%. 3. Bayesian skyline plot (BSP) analyses supported a continuous population expansion for the total and for the Ecuadorian *T. pinchaque* samples throughout the last 100,000 years. The highest increases in females occurred in the last 25,000 and 10,000 years, respectively. In contrast, the Colombian sample yielded a more constant population size in the last 100,000 years, with a strong decrease in the last 5000 years. 4. Phylogenetic trees did not yield comprehensive clades reflecting geographical distribution. There were only some small clades with specimens of related geographical areas. 5. The phylogenetic trees also showed reciprocal monophyly between *T. terrestris* and *T. pinchaque* with the data supporting *T. kabomani* as a clade within *T. terrestris*.

In the current work, we expanded our molecular analyses of *T. pinchaque* to deepen our understanding of this species’ genetic structure and to determine the relationship between *T. pinchaque* and the other species of Neotropical tapirs (including the alleged *T. kabomani*). In fact, it was shown that the phylogenetic relationships of *T. pinchaque* with other tapir species are essential to demonstrate the inexistence of *T. kabomani* as a full species [7,8,9].

To carry this out, we sequenced the complete mitogenome of 44 specimens of this species, as well as the nuclear *RAG1-2* genes (recombination-activating gene 1 and gene 2) of 12 specimens, the nuclear *IRBP* gene (interphotoreceptor retinoid-binding protein) of 11 specimens, and the nuclear *BRCA1* gene (breast and ovarian cancer susceptibility gene 1) of 10 specimens.

*RAG1* and *RAG2* genes encode the components of the recombinase involved in the recombination of immunoglobin and T-cell receptor genes and appear as conserved single copies in all examined vertebrates [27,28]. Recombination-activating genes are expressed only in the nucleus of developing B and T lymphocytes, where the *RAG1* and *RAG2* proteins act synergistically to make double-stranded breaks at specific recognition sequences, initiating recombination of variable, diverse, and joining gene segments [29]. Nucleotide sequence data from *RAG1* have previously been analyzed in several vertebrate phylogenetic studies [30,31].

*IRBP* is a single-copy nuclear gene, in all taxa so far examined, with no reported pseudogenes. This gene consists of four exons spanning 9.5 kb of genomic DNA [32]. The gene product is a large (1230 amino acids) secreted glycolipoprotein that is primarily found in the extracellular matrix between the retinal pigment epithelium and the neural retina of the eye. It is believed to be involved in the extracellular transfer of retinoids between the retina and pigment epithelium during light and dark adaptation [32]. Nucleotide sequences from *IRBP* exon 1 were among the first molecular character data to provide convincing support for mammalian superordinal relationships [33,34,35], as well as in diverse studies focused on lower-level relationships in mammals [36].

The *BRCA1* gene consists of 22 coding exons distributed over 100 kb of genomic DNA on the long arm of human chromosome 17 [37]. This gene is responsible for most hereditary forms of breast cancer and accounts for as many as 10% of all breast cancer cases in humans [38]. Because the products of this gene play a critical role in key cellular processes such as DNA repair, cell cycle control, and transcriptional regulation, it is clear why mutations could be so detrimental. Given its indispensable functions in maintaining the integrity of the genome, one might expect the strict evolutionary conservation of *BRCA1* over time. Nevertheless, contrary to this line of reasoning, several authors have documented the rapid evolution of *BRCA1* in mammals [39,40,41]. Rapid evolution occurs when a gene experiences positive natural selection for new, advantageous mutations that arise in a population. Sequences from *BRCA1* exon 11 have been used for several mammalian phylogenetic studies, with important results [42,43,44,45].

Taking all of this into consideration, the main aims of the current research are as follows: (1) To determine the phylogenetic relationships between *T. pinchaque* and the other Neotropical tapir species (including the alleged *T. kabomani*) using mitogenomes and three types of nuclear genes *(RAG1-2*, *IRBP*, and *BCRA1*); (2) To clarify whether *T. kabomani* is really a full and differentiated tapir species; (3) To estimate the levels of genetic diversity for *T. pinchaque* using mitogenomes and these three nuclear genes; (4) To detect some kind of spatial genetic structure for *T. pinchaque* using mitogenomes and these three nuclear genes; and (5) To detect possible demographic change throughout the natural history of *T. pinchaque* using mitogenomes and these three nuclear genes.

## 2. Material and Methods

### 2.1. Samples

**Figure 1:**
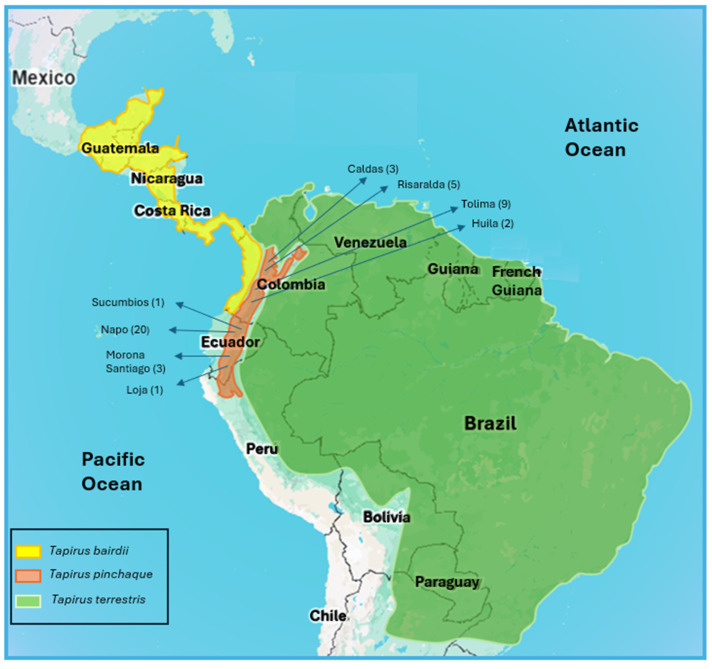
Map with the geographical distribution of the three tapir species in the Neotropics. Within the geographical distribution of *Tapirus pinchaque*, the four departments (Colombia) and four provinces (Ecuador), where 44 specimens were sampled, are shown. In parenthesis, the numbers of specimens sampled in each department or province.

We analyzed 44 *T. pinchaque* samples from Colombia and Ecuador with high-quality DNA for complete mitogenomes (16,712 base pairs, bp). The geographical origins of these 44 specimens can be seen in Figure 1 and Table 1. Additionally, 94 samples of *T. terrestris* (including 5 samples of *T. kabomani*) and 18 samples of *T. bairdii* were also sequenced for complete mitogenomes. Furthermore, 12 specimens of *T. pinchaque* were sequenced at the nuclear *RAG1-2* genes (as well as 21 *T. terrestris*, including 5 *T. kabomani*, and 12 *T. bairdii*), 11 specimens of *T. pinchaque* at the nuclear *IRBP* gene (as well as 34 *T. terrestris*, including 6 *T. kabomani*, and 14 *T. bairdii*), and 10 specimens at the nuclear *BRCA1* gene (as well as 16 *T. terrestris*, including 5 *T. kabomani*, and 16 *T. bairdii*). *T. pinchaque* samples were obtained from pieces of skin and bones (specimens which had been previously hunted by peasant or indigenous communities) and captive animals in zoos (with geographical origins; hairs and blood) as well as from tapirs captured for research (radio tracking) both in Ecuador (A. Castellanos) and in Colombia (F. Kaston) (blood). The samples for the remainder tapir species were also obtained from pieces of skin and bones (previously hunted by peasant or indigenous communities), captive animals in zoos or in indigenous communities (with geographical origins; hairs and blood), and pieces of skin from museums (with geographical origins).

The six *T. kabomani* samples came from the Aripuana River (Brazilian Amazon; three specimens), Amacayacu National Park (Colombian Amazon; one specimen), the Mazan River in the Loreto Department (Peruvian Amazon; one specimen), and the Napo River in Napo Province (Ecuadorian Amazon; one specimen). The mt *COI-COII-Cyt-b* genes of these samples, as well as those of the two Brazilian and the two Colombian samples of *T. kabomani* previously reported [4], formed a homogenous clade and therefore all these samples were analyzed, considering them as *T. kabomani*. In fact, one of the Colombian samples previously used by [4] is the same Colombian sample we analyze herein.

On the other hand, our *T. pinchaque* sample was highly representative of the major part of the geographical distribution of this species (Central Colombian Andean cordillera and the entire Ecuador). Nevertheless, two areas where this species lives were not sampled: the Eastern Colombia Andean cordillera and a very small population in northern Peru. In the Andean areas where *T. pinchaque* currently exists, there is no evidence of the presence of *T. terrestris* or *T. bairdii*. Thus, potential hybridization between *T. pinchaque* and other species of tapirs is highly unlikely nowadays. Both *T. bairdii* (living in lowlands from Mexico, Central America, and Pacific Colombia) and *T. terrestris* (living manly in the Amazon and other lowlands of other non-Andean areas of South America) are outside from the restricted Andean distribution of *T. pinchaque*.

The permissions to capture and obtain samples of tapirs were given by the Ministerio de Desarrollo Sostenible y Planificación, Dirección General de Biodiversidad from Bolivia (DGB/UVS No 477/03; approval date 27 May 2003) and CITES Bolivia (B09118259 and B09118514), by 065-2024-EXP-CM-DBI/MAATE, No. 048.October-2018-MEPN, MAE-DNB-CM-2019-0126, and MAATE-ARSFC-2022-2583 (Ecuador), Ministerio de Producción (No 402-2003 PRODUCE/DNEPP) (Peru), and Colección de Mamíferos del Instituto Humboldt IAvH (Registro Nacional de Colecciones, No. 003).

### 2.2. Molecular Procedures

DNA was extracted and isolated from blood and muscle samples using the QIAamp DNA Mini Kit (Qiagen, Inc. Germantown, MD, USA). Amplifications for mitogenomes were carried out using the LongRange PCR Kit (Qiagen, Inc. Germantown, MD, USA) with a final volume reaction of 25 μL. The reaction mixture was composed of an 80–200 ng DNA template, 2 units of Long-Range PCR Enzyme, 3 μL of 10× LongRange PCR Buffer, 4 μL (15 pmol) of each primer, and 2 μL of 10 mM dNTPs. The cycling conditions were 95 °C for 3 min, followed by 50 cycles denaturing at 95 °C for 20 s, primer annealing at 53–58 °C (with a decrease of 0.1 °C every cycle) for 30 s, and extension at 72 °C for 10 min. This was followed by 30 cycles of denaturing at 95 °C for 20 s, annealing at 48–53 °C for 30 s, and extension at 72 °C for 5 min, with a final extension at 72 °C for 10 min. All amplifications, including positive and negative controls, were checked onto 2% agarose gels under a Hoefer UV Transilluminator (Hoefer Inc. Bridgewater, MA, USA). Both mtDNA strands were sequenced directly with a Big Dye Terminator version 1.1, version 3.1 in an automated 3100 Genetic Analyzer (Applied Biosystems, Foster City, CA, USA). We used four sets of primers [8] to generate overlapping amplicons from around 3300 bp to 5000 bp in length for a total length of 16,712 bp. MtDNA data, due to potential nuclear insertions (numts), can complicate phylogenetic analyses and interpretations. However, the amplicons obtained allowed us to carry out a quality test for genome circularity [46,47], and to determine the inexistence of these numts in our data.

The *RAG1* sequences were a fragment of the exon 1 of 1159 bp in length, using the RAG1F1705 and RAG1R2864 primers [48]. The *RAG2* sequences corresponded to 1187 bp using the RAG2F1-110 and RAG2R4-1297 primers [49]. As *RAG1* and *RAG2* are closely linked, we summed the sequences of these genes (a total of 2346 bp). The *IRBP* sequences belonged to a fragment of exon 1 of 1314 bp in length using the IRBPF217 and IRBPR1531 primers [33,34]. The *BCRA1* sequences were fragments from exon 11 equaling 2422 bp in length using the MBF1 and MBR6 primers [50]. Thus, a total nuclear gene length of 6082 bp was analyzed.

The PCR mix for each nuclear gene contained 10 mM Tris (pH 8.3), 50 mM KCl, 0.01% gelatin, 0.1% Triton X-100, 2.5 mM MgCl_2_, 0.2 mM dNTP mix, 0.05 µM of each primer (1 pmol of each primer per reaction), 0.5 units of Amplitaq DNA polymerase (Applied Biosystems, Foster City, CA, USA), and 0.2–0.7 µg of template total genomic DNA in a total volume of 20 µL. The cycling parameters for the PCR for each nuclear gene were as follows: for *RAG1*, a cycle of denaturation at 95 °C for 3 min and 30 cycles of denaturation at 95 °C for 30 s, annealing at 57 °C for 30 s, and extension at 72 °C for 1 min. For *RAG2*, the PCR cycling was performed using a ‘‘touchdown’’ program, with a 30 s denaturation at 95 °C, annealing for 60 s at 58, 56, 54, and 52 °C (two cycles at each temperature), and 90 s extension at 72 °C, for a total of 8 cycles; this regime was followed by 27 cycles of 30 s at 95 °C, 1 min at 50 °C, and 90 s at 72 °C, with a final extension of 72 °C for 5 min. For the *IRBP* gene, during PCR cycling, denaturation occurred at 95 °C for 10 min, followed by 27 cycles at 95 °C for 25 s, 58 °C for 20 s, and 72 °C for 1 min, with a final extension at 72 °C for 10 min. For *BCRA1*, PCR cycling consisted of initial denaturing at 94 °C for 15 min, 45 cycles of 94 °C for 20 s, 55 °C for 20 s, and 72 °C for 30 s, with a final extension at 72 °C for 7 min.

Agarose (2%) gels were used to visualize the amplified products. Polymerase chain reaction products were purified using a QIAquick PCR Purification Kit (Qiagen Inc.) with either polyethylene glycol or ExoSAP-IT (Affymetrix, Santa Clara, CA, USA). DNA sequencing was performed for both light and heavy strands with a Big Dye Terminator version 1.1, version 3.1 in an automated 3100 Genetic Analyzer (Applied Biosystems, Foster City, CA, USA).

### 2.3. Mathematical Population Analyses

#### 2.3.1. Phylogenetics and Population Genetics Procedures

jModeltest v2.0 software [51] was applied to determine the best evolutionary mutation model for the sequences analyzed for each individual gene (nuclear and mitogenomes), for different partitions (mitogenomes), and for all the concatenated sequences (mitogenomes). Akaike information criterion (AIC) [52] and the Bayesian information criterion [53] were used to determine the best evolutionary nucleotide model for the global mitogenome tree and for each one of the nuclear genes studied in the tapirs.

Phylogenetic trees were constructed by using maximum likelihood trees (ML). The ML trees were obtained using the RAxML v8.2.X software [54] implemented in CIPRES Science Gateway [55]. The different evolutionary nucleotide models we used to search for the ML tree reflect the specific nuclear genes and mitogenomes we analyzed. We estimated support for nodes using the rapid-bootstrapping algorithm (−f a−x option) for 1000 non-parametric bootstrap replicates [56].

To reconstruct the possible relationships among the haplotypes (mt) and alleles (nuclear) of the tapir species analyzed and to estimate possible divergence times among these haplotypes and alleles, we constructed four median joining networks (MJNs) (one for mitogenomes, and three for the nuclear genes analyzed) using Network v4.6.0.1 software (Fluxus Technology Ltd. Stanway, Colchester, England) [57]. Additionally, the ρ statistic [58] and its standard deviation [59] were estimated and transformed into years. The ρ statistic is unbiased and highly independent of past demographic events. This approach is named “borrowed molecular clocks” and uses direct nucleotide substitution rates inferred from other taxa [60]. We used an evolutionary rate for mitogenomes of 1.12% as per [61], which represented one mutation each 5343 years for the 16,712 bp analyzed. For the nuclear *RAG1-2* genes, we used an evolutionary rate of 0.2% as per [62] which represented one mutation each 213,129 years for the 2346 bp analyzed; meanwhile, for the nuclear *IRBP* gene, we used 0.12% as per [61] (representing one mutation each 679,496 years for the 1314 bp analyzed), and for the nuclear *BCRA1* gene, we used 0.14% as per [63] (representing one mutation each 277,910 years for the 2422 bp analyzed). Network analyses can be more useful in reconstructing evolutionary history within species or among very related species than bifurcating trees, as is the case within the genus *Tapirus*.

#### 2.3.2. Genetic Diversity Statistics

We used the following statistics to determine the genetic diversity for the overall sample of *T. pinchaque* as well as for *T. terrestris* and *T. bairdii*: number of haplotypes (H) for mitogenomes and number of alleles (NAs) for nuclear genes, haplotype diversity (H_d_) for mitogenomes and expected heterozygosity for nuclear genes (H_e_), and nucleotide diversity (π) for both types of markers. These genetic diversity statistics were calculated with the programs DNAsp 5.1 [64] and Arlequin 3.5.1.2 [65].

#### 2.3.3. Spatial Genetic Structure in *T. pinchaque*

Four methods were used for *T. pinchaque* for both mitogenomes, and the three nuclear gene datasets studied. First, the Mantel test [66] was used to detect possible relationships between a genetic matrix of the *T. pinchaque* specimens analyzed (Kimura 2P genetic distance) and the geographic distance matrix among the specimens analyzed throughout Colombia and Ecuador. In this study, Mantel’s statistic was normalized [67], which transforms the statistic into a correlation coefficient. Second, a spatial autocorrelation analysis utilized the A_y_ statistic [68] for each distance class (DC). Ay can be interpreted as the average genetic distance between pairs of individuals that fall within a specific DC, with a value of 0 when all individuals within a DC are genetically identical and a value of 1 when all individuals within a DC are completely dissimilar. The probability for each DC was obtained using 10,000 randomizations. For the analysis of the mitogenomes, six DCs were defined with each distance class of the same size; meanwhile, for the nuclear genes, four DCs were defined with each distance class of the same size too. These analyses were carried out with AIS 1.0 software [68]. The third procedure used was a genetic landscape interpolation analysis (GLIA), which was performed by means of the “inverse distance weighted” method [68], to view the spatial genetic structure of the data in three-dimensional space for the mitogenomes and the three nuclear gene datasets.

Additionally, for all the molecular markers used with *T. pinchaque*, we conducted a Bayesian analysis of population structure with the software BAPS v6.0 [69], using mixed and unmixed models to group genetically similar individuals into panmictic genetic clusters. Calculations were performed with the number of k clusters varying from 2 to 11. Five replicates were carried out for each k value.

#### 2.3.4. Possible Historical Demographic Changes in *T. pinchaque*

Different procedures were used to detect possible historical demographic changes in the overall sample of *T. pinchaque*. First, we used the Fu and Li’s D* and F* tests [70], Fu’s F_S_ statistic [71], Tajima’s D test [72], and R2 statistic [73] to determine possible demographic changes. Both 95% confidence intervals and probabilities were obtained with 10,000 coalescence permutations. Second, a mismatch distribution (pairwise sequence differences) was obtained [74,75]. We used raggedness (*rg*) to determine the similarity between the observed and theoretical curves. These demographic analyses were carried out using DNAsp v5.1 [64] and Arlequin v3.5.1.2 [65]. Third, we used the coalescence-based BSP to estimate demographic changes in effective numbers. BSP analysis was performed in BEAST v2.4.3 using the empirical base frequencies and a strict molecular clock [76,77]. We applied jModelTest2 to evaluate the best substitution models. Additionally, we assumed a substitution rate of 7.28 × 10^−8^ substitutions per site and per year (mitogenomes), 2.5 × 10^−8^ substitutions per site and per year (nuclear *RAG1-2*), 1.02 × 10^−8^ substitutions per site and per year (nuclear *IRBP*), and 7.27 × 10^−9^ substitutions per site and per year (nuclear *BRCA1*) in order to obtain the time estimates in years as well as with kappa with log-normal (1, 1.25), and a skyline population size with uniform (0, infinite; initial value 80). We conducted a total of five independent runs of 40 million Markov chain Monte Carlo (MCMC) iterations following a burn-in of 10% of iterations, logging every 10,000 iterations. We selected a stepwise (constant) Bayesian skyline variant with maximum time as the upper 95% high posterior density (HPD). The total height of the tree was determined by treeModel.rootHeight. The convergence of the analysis was assessed by checking the consistency of the results over five independent runs. For each run, we used the software Tracer v1.7 to inspect the trace plots for each parameter to assess the mixing properties of the MCMCs and to estimate the ESS value for each parameter. Runs were considered as having converged if they displayed good mixing properties and if the ESS values for all parameters were greater than 200. We discarded the first 10% of the MCMC steps as a burn-in and obtained the marginal posterior parameter distributions from the remaining steps using Tracer v1.7. To test whether the inferred changes of effective numbers over time were significantly different from a constant population size null hypothesis, we compared the BSP obtained with the ‘Coalescent Constant Population’ model (CONST) implemented in BEAST v2.4.3 using Bayes Factors. Therefore, we conducted five independent CONST runs using 40 million MCMC iterations after a burn-in of 10%, logging every 10,000 iterations. We assessed the proper mixing of the MCMC and ensured that ESSs were greater than 200. We then used the Path sampler package in BEAST v2.4.3 to compute the log of marginal likelihood (logML) of each run for both BSP and CONST. We set the number of steps to 100 and used 40 million MCMC iterations after a burn-in of 10%. Bayes factors were computed as twice the difference between the log of the marginal likelihoods (2[Log ML(BSP)–Log ML(CONST)]) and were performed for pairwise comparisons between BSP and CONST runs. As recommended, Bayes factors greater than 6 were considered as strong evidence to reject the null hypothesis of constant effective numbers throughout time. Nevertheless, all these demographic procedures have several caveats. Selection can affect the effective population size, reducing the effective number for a time and increasing the coalescence rate later [78]. The same occurs with small changes in the mutation rate (μ), which can greatly affect the effective number and, in turn, estimated divergence time [79].

Finally, a birth–death skyline contemporary procedure [80], using BEAST v2.4.3, was applied for *T. pinchaque* for mitogenomes and for the nuclear *RAG1-2* genes. The main priors we used were as follows: uninfectious rate (Log Normal; initial = [1.0] [0.0, infinite]), gamma shape (exponential; initial = [1.0] [−infinite, infinite]), birth–death skyline origin (uniform; initial = [1000] [0.0, infinite], to analyze the last 250,000 years for mitogenomes and 500,000 years for the nuclear *RAG1-2* genes and dimension = 10), reproductive number (Log Normal; initial = [2.0] [0.0, infinite]), and rho (Beta; initial = [0.01] [0.0, 1]). The chain length was 20 million and there was a burn-in of 10%. If *R_e_* (reproductive number) > 1, the number of haplotypes increases; if *R_e_* = 1, the number of haplotypes remains constant; and if *R_e_* < 1, the number of haplotypes decreases.

## 3. Results

### 3.1. Phylogenetics of the Neotropical Tapirs

The complete mitogenome (16,712 bp) for 44 *T. pinchaque* specimens showed that the best nucleotide substitution models were HKY + G + I (for BIC; 180,451.035) and TN93 + G + I (for AICc; 130,089.624), respectively. For the three nuclear loci, the best nucleotide substitution models were as follows: For *RAG1–RAG2*, they were HKY + G (for BIC; 13,301.165) and TN93 + G + I (for AICc; 12,410.701), whereas for *IRBP* they were T92 (for BIC; 3778.221), and HKY (for AICc; 2768.876), and for *BCRA1,* they were HKY + G (for BIC; 4085.525) and TN93 + G (for AICc; 3348.219), respectively.

**Figure 2:**
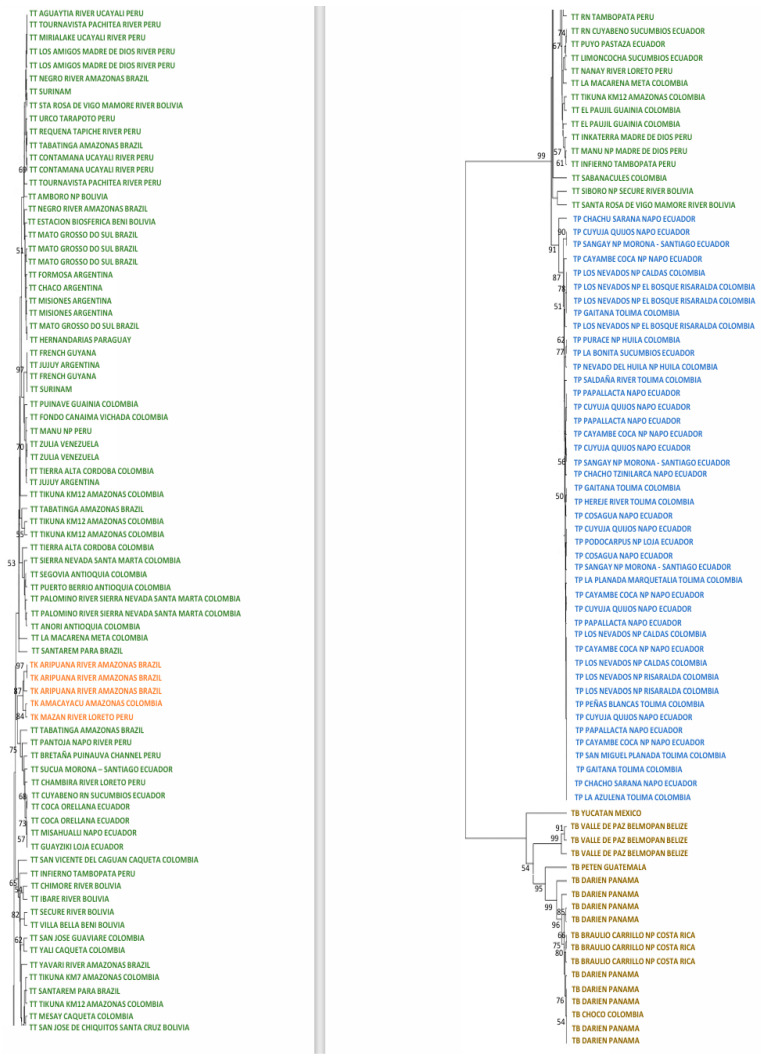
Maximum likelihood tree analyzing the mitogenomes of 44 *T. pinchaque* (blue), 89 *T. terrestris* (green), five “*T. kabomani*” (orange), and 18 *T. bairdii* (brown). In nodes, percentages of bootstraps.

The ML tree of the mitogenome dataset (Figure 2) for the Neotropical tapirs showed that the ancestor of *T. bairdii* was the first to diverge. Within this clade, the most divergent specimen was the one from Mexico. Three specimens from Belize conformed a strong group (bootstrap: 99%), meanwhile, one specimen from Guatemala was also differentiable (95%). The remaining specimens from Costa Rica, Panama, and Colombia formed a unique, significant group (99%).

Later, the split of the ancestors of *T. terrestris* (99%) and *T. pinchaque* (91%) appeared. Multiple clusters appeared within *T. terrestris*, many of them with low bootstraps and some of them with elevated bootstraps. Likely, some of these clusters have determined geographical origins, whereas others have no geographical signal (diverse specimens from different geographical and distant areas). For example, one robust cluster (97%) grouped three specimens from French Guiana and Surinam (same geographical area), but it also enclosed a specimen from Jujuy (Argentina), although the origin of this specimen (a captive one) was from northern South America. Another cluster with a clear geographical origin had a medium bootstrap (53%) and it was integrated by specimens from northern Colombia (Antioquia, Córdoba, and Magdalena Departments). Another cluster with a clear geographical origin and elevated bootstrap (75%) comprised specimens from Ecuador and western Amazon (Peru and Brazil). The most outstanding cluster inside *T. terrestris* may be the one composed of five specimens with mitochondrial characteristics of *T. kabomani* (87%) from Colombia, Brazil, and Peru. The current results, together with those previously obtained [7,8,9], clearly showed that *T. kabomani* is not a fully differentiated species from *T. terrestris*. It is a differentiable molecular lineage, but within *T. terrestris*.

Within *T. pinchaque*, the most differentiated specimen was the one from Chaco, Sarañan (Napo Province, Ecuador). Two additional small and significant clusters were one (90%) integrated by two Ecuadorian specimens (Cuyuja, Quijos, Napo Province, and Sangay NP, Morona-Santiago Province) and others (77%) composed of three specimens (two from Huila Department in Colombia, and one from Sucumbios Province in northern Ecuador). A third small cluster (78%) was integrated by some of the specimens sampled in the most northern distribution area of this species (Los Nevados National Park in Caldas and Risaralda Departments as well as one specimen in the Tolima Department, all of them in Colombia). The remaining specimens (Colombian and Ecuadorian ones) were intermixed in clusters of low significance. Therefore, we found a small genetic structure for *T. pinchaque* throughout its distribution, except for the most northern and peripatric distribution in Colombia and some local patches throughout Ecuador.

**Figure 3:**
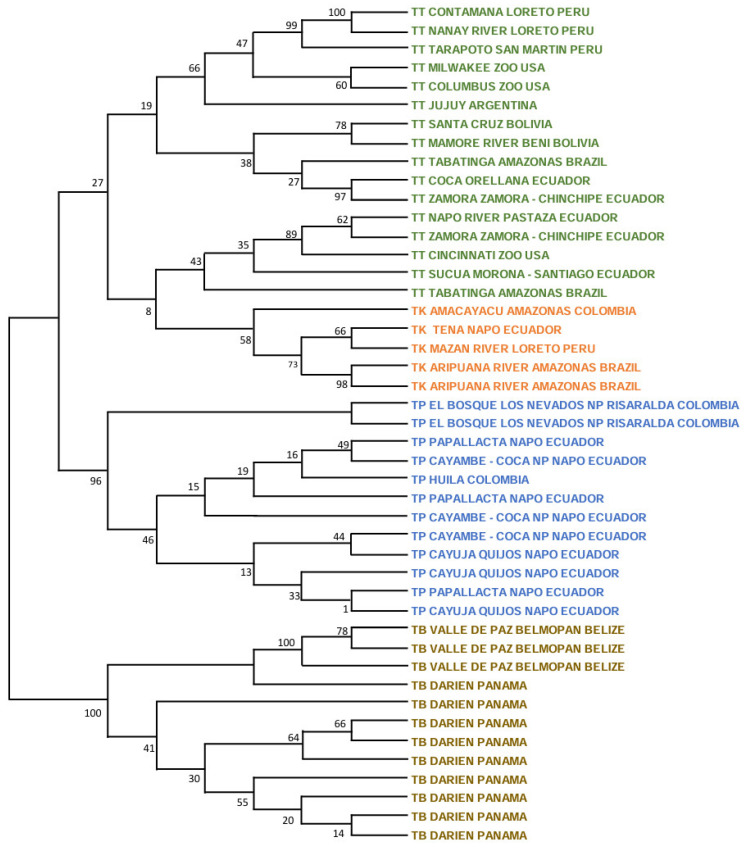
Maximum likelihood tree analyzing the nuclear *RAG 1-2* genes of 12 *T. pinchaque* (blue), 16 *T. terrestris* (green), five “*T. kabomani*” (orange), and 12 *T. bairdii* (brown). In nodes, percentages of bootstraps.

The ML tree with the nuclear *RAG1-RAG2* genes showed the following picture (Figure 3). For *T. terrestris*, many small significant clusters were present, including in two Peruvian specimens (100%; Contamaná and Nanay River, Loreto Department), two Bolivian specimens (78%: Beni and Santa Cruz Departments), two Ecuadorian specimens (97%; Orellana and Zamora-Chinchipe Provinces), and three Ecuadorian and one US zoo specimens (89%; Pastaza and Zamora-Chinchipe Provinces in Ecuador, and Cincinnati Zoo). The five specimens of *T. kabomani* formed a clade with a medium bootstrap (58%), clearly more related to *T. terrestris* than to any other Neotropical tapir taxa. In reference to *T. pinchaque*, this tree showed that two specimens for the Los Nevados NP in the Colombian Department of Risaralda are those most differentiated (84%), whereas the remaining specimens did not show any strong cluster.

**Figure 4:**
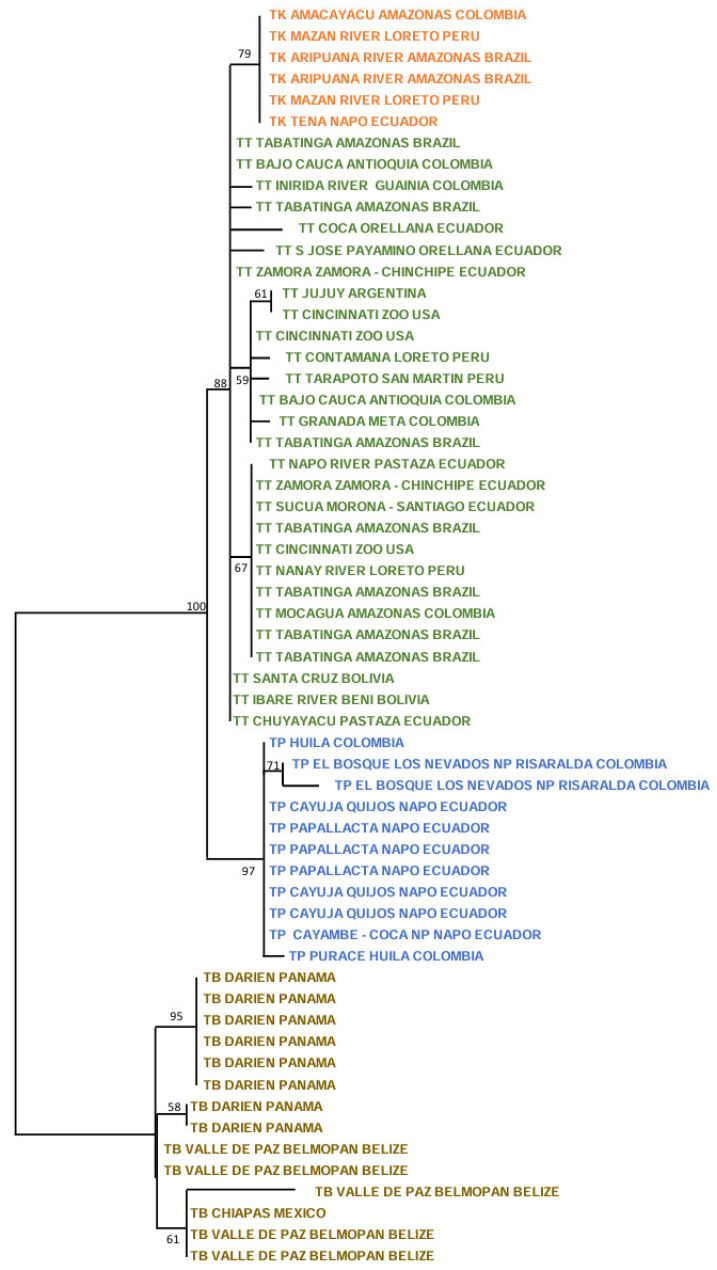
Maximum likelihood tree analyzing the nuclear *IRBP* gene of 11 *T. pinchaque* (blue), 28 *T. terrestris* (green), 6 “*T. kabomani*” (orange), and 14 *T. bairdii* (brown). In nodes, percentages of bootstraps.

The ML tree for *IRBP* showed that inside *T. terrestris* (88%), there were three significant clusters (Figure 4). One was composed of six specimens of *T. kabomani* (79%), while another (59%) was composed by specimens of diverse origins, including US zoos (Argentina, Brazil, Colombia, and Peru). The third cluster (67%) was integrated by specimens from Brazil, Colombia, Ecuador, and US zoos. *T. kabomani* was again a significant cluster within *T. terrestris*. For *T. pinchaque* (97%), we observed, that the same two Colombian specimens from Los Nevados NP of the previous nuclear ML tree were the most differentiated (71%). The remaining specimens did not show any significant cluster.

**Figure 5:**
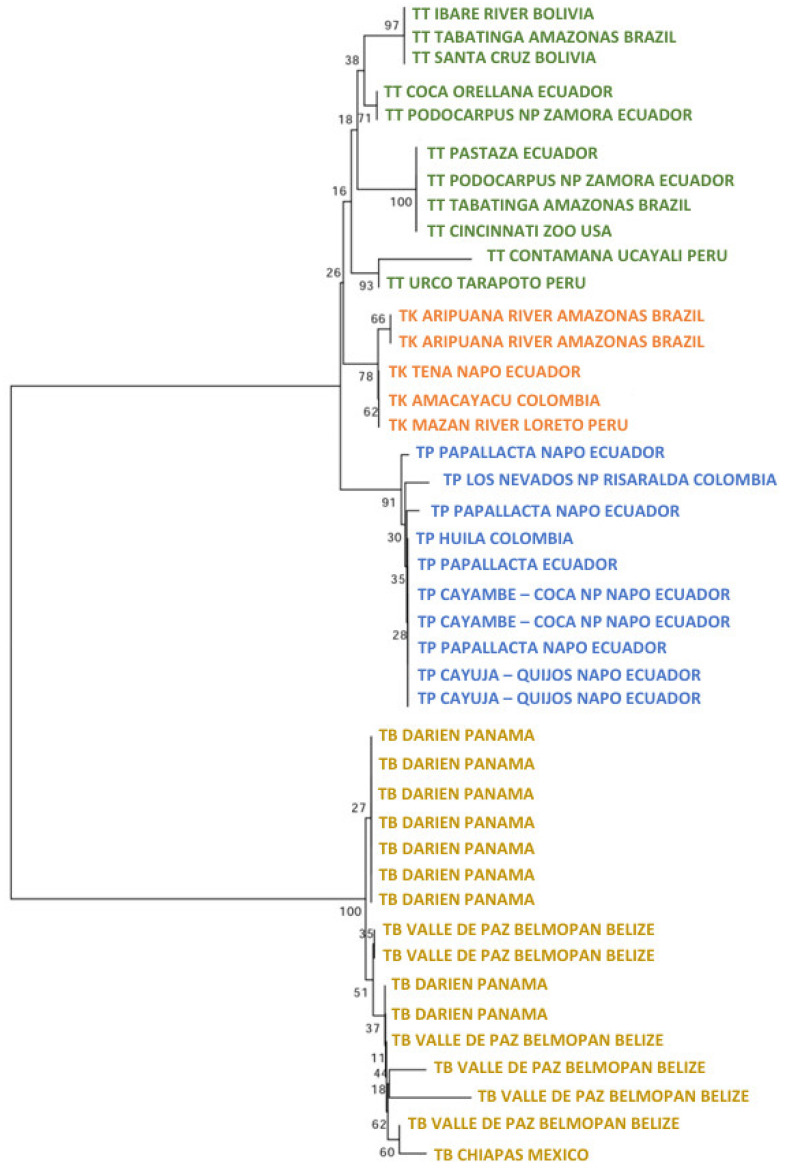
Maximum likelihood tree analyzing the nuclear *BRCA1* of 10 *T. pinchaque* (blue), 11 *T. terrestris* (green), five “*T. kabomani*” (orange), and 16 *T. bairdii* (brown). In nodes, percentages of bootstraps.

The ML tree for *BRCA1* (Figure 5) showed five significant clusters inside *T. terrestris*. The first (97%) was composed of specimens from Bolivia and Brazil. The second cluster (71%) had two specimens from Ecuador (Orellana and Zamora-Chinchipe Provinces). The third (100%) had specimens from Brazil and Ecuador plus one from a US zoo. The fourth (93%) contained two Peruvian specimens (Loreto and San Martín Departments) and the fifth cluster (78%) contained five specimens of *T. kabomani*. Once more (as in the previous mitogenome and nuclear trees), *T. kabomani* is a sub-clade inside *T. terrestris,* re-affirming the improbability of *T. kabomani* as a full and real species. In the case of *T. pinchaque*, the two most differentiated specimens were from Papallacta (Napo Province, Ecuador) and Los Nevados NP (like the previous analyses). The remaining specimens yielded no structure. For all four molecular markers, the genetic structure (and the number of significant clusters) of *T. pinchaque* was considerably smaller than in *T. terrestris*.

**Figure 6:**
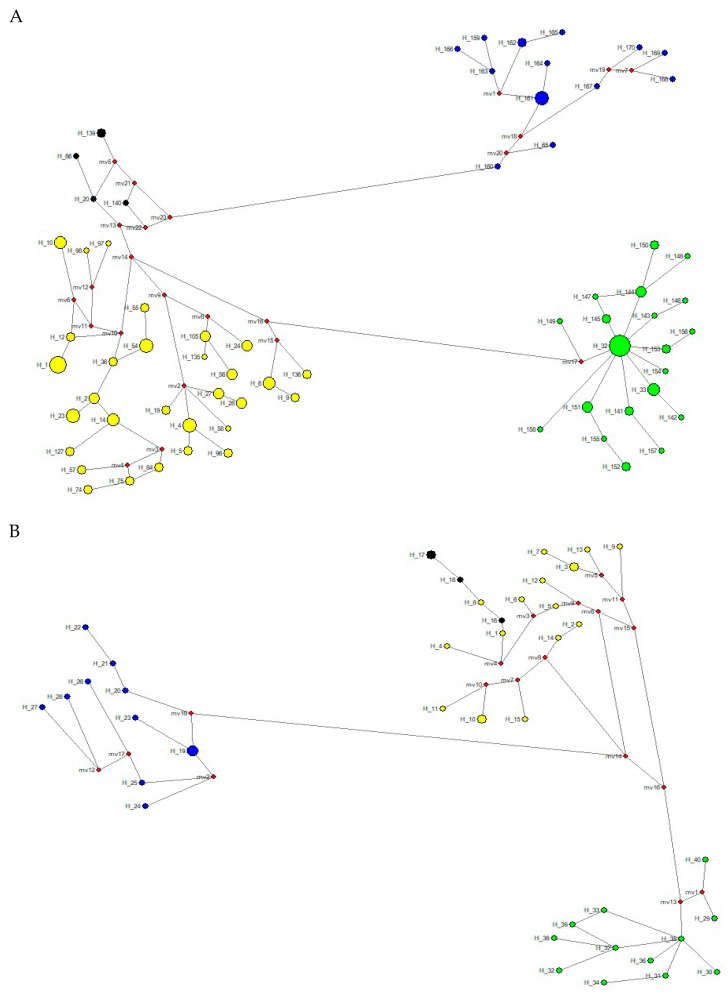

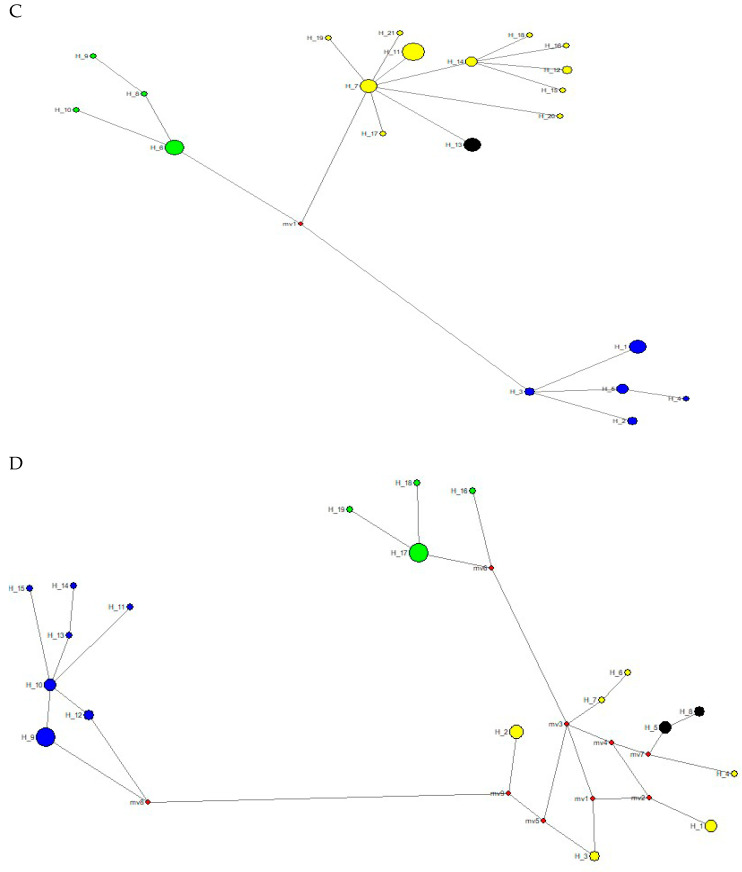
(**A**) Median-joining network (MJN) with haplotypes identified in 156 specimens of tapirs (44 *T. pinchaque*, 89 *T. terrestris*, 5 “*T. kabomani*”, and 18 *T. bairdii*) sampled in the Neotropics for overall mitogenomes. (**B**) Median-joining network (MJN) with haplotypes identified in 45 specimens of tapirs (12 *T. pinchaque*, 16 *T. terrestris*, 5 “*T. kabomani*”, and 12 *T. bairdii*) sampled in the Neotropics for the nuclear *RAG 1-2* genes. (**C**) Median-joining network (MJNs) with haplotypes identified in 59 specimens of tapirs (11 *T. pinchaque*, 28 *T. terrestris*, 6 “*T. kabomani*”, and 14 *T. bairdii*) sampled in the Neotropics for the nuclear *IRBP* gene. (**D**) Median-joining network (MJN) with haplotypes identified in 42 specimens of tapirs (10 *T. pinchaque*, 11 *T. terrestris*, 5 “*T. kabomani*”, and 16 *T. bairdii*) sampled in the Neotropics for the nuclear *BRCA1* gene. Blue = *T. bairdii*; yellow = *T. terrestris*; black = “*T. kabomani*”; green = *T. pinchaque*. Small red circles indicate missing intermediate haplotypes.

The MJN procedure (Figure 6A) with the mitogenome dataset showed a temporal split that occurred between *T. bairdii* and *T. terrestris* at around 5.71 ± 0.11 mya. The temporal division between *T. terrestris* and *T. pinchaque* was estimated to have occurred around 1.33 ± 0.170 mya, while the split between *T. terrestris* and *T. kabomani* was dated to approximately 0.578 ± 0.201 mya. The haplotype diversification within *T. bairdii* was estimated to have occurred around 1.66 ± 0.179 mya, whereas within *T. terrestris* and within *T. pinchaque,* the temporal splits were estimated to have occurred around 1.01 ± 0.176 mya and 0.411 ± 0.171 mya, respectively.

The MJN procedure (Figure 6B) with the *RAG1-RAG2* genes showed a temporal split between *T. bairdii* and *T. terrestris* around 8.08 ± 0.039 mya. The temporal division between *T. terrestris* and *T. pinchaque* was estimated to have occurred around 4.44 ± 0.158 mya, while the split between *T. terrestris* and *T. kabomani* was dated to around 1.03 ± 0.285 mya. The MJN procedure (Figure 6C) with the *IRBP* gene showed a temporal split between *T. bairdii* and *T. terrestris* to have occurred around 4.55 ± 0.201 mya. The temporal division between *T. terrestris* and *T. pinchaque* was estimated to have occurred around 0.541 ± 0.187 mya, while the split between *T. terrestris* and *T. kabomani* was dated to around 0.189 ± 0.134 mya. The MJN procedure (Figure 6D) with the *BRCA1* gene showed a temporal split to have occurred between *T. bairdii* and *T. terrestris* around 6.62 ± 0.220 mya. The temporal division between *T. terrestris* and *T. pinchaque* was estimated to have occurred around 1.34 ± 0.167 mya, while the split between *T. terrestris* and *T. kabomani* was dated to around 0.687 ± 0.162 mya.

If we take an approximate average of these temporal estimates (considering the different nucleotide substitution rates for each one of the genes used), the temporal split between *T. bairdii* and *T. terrestris* was 6.24 ± 0.191 mya. The temporal division between *T. terrestris* and *T. pinchaque* was estimated to have occurred around 1.91 ± 0.171 mya, while the split between *T. terrestris* and *T. kabomani* was dated around 0.621 ± 0.195 mya. Clearly, *T. kabomani* split from the other haplogroups of *T. terrestris* much more recently than the ancestor of *T. pinchaque*. This result again reaffirms the fact that *T. kabomani* is not a full species, which is in contrast with what was previously suggested [4,5].

### 3.2. Genetic Diversity in the Neotropical Tapirs

Diverse genetic diversity statistics were found in the three Neotropical tapir species. Two results were particularly noteworthy. First, *T. pinchaque* is the species with the lowest levels of genetic diversity correlated with its small geographical distribution. For mitogenomes, *T. pinchaque* had a H_d_ of 0.900 ± 0.003 and a π of 0.0016 ± 0.003. These values were much higher for *T. terrestris* (H_d_ = 0.994 ± 0.0001 and π = 0.0137± 0.00005) and *T. bairdii* (H_d_ = 0.928 ± 0.005 and π = 0.0218 ± 0.0001). If we take the average median for the three nuclear molecular markers used for the three Neotropical tapir species, the situation is about the same as that of the mitogenomes. *T. pinchaque* presented an average H_e_ of 0.675 ± 0.129 and π = 0.0014 ± 0.0005, whereas the values were much greater in *T. terrestris* (H_e_ = 0.914 ± 0.033 and π = 0.0089 ± 0.0015) and *T. bairdii* (H_e_ = 0.842 ± 0.077 and π = 0.0061 ± 0.0018). Thus, *T. pinchaque* showed the lowest values of genetic diversity for both kinds of DNA, and, in contrast to the nuclear genes we sequenced, mitogenomes always showed higher levels of genetic diversity for the three tapir species.

### 3.3. Spatial Genetic Patterns in T. pinchaque

**Figure 7:**
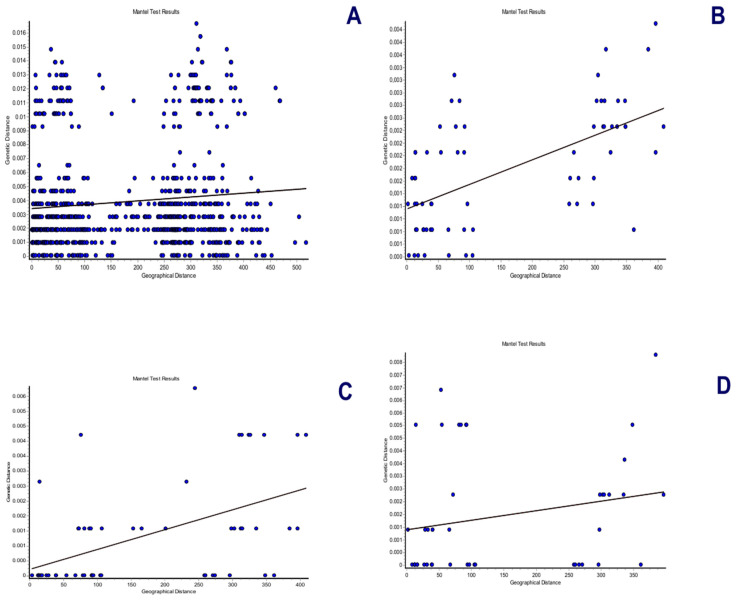
Mantel tests between the geographic and genetic distances for the specimens of Andean or mountain tapirs (*T. pinchaque*) sampled in Colombia and Ecuador. (**A**) For overall mitogenomes; (**B**) For the nuclear *RAG 1-2* genes; (**C**) For the nuclear *IRBP* gene; and (**D**) For the nuclear *BRCA1* gene.

**Figure 8:**
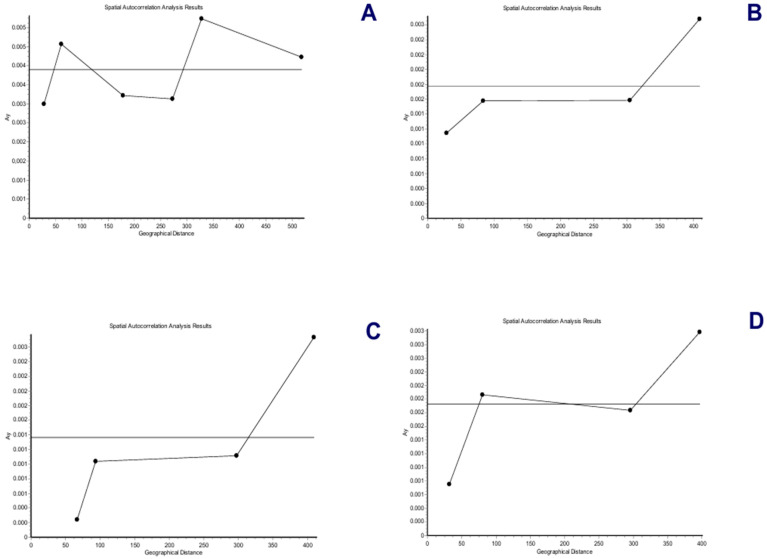
Correlograms with the A_y_ statistic and six distance classes (mitogenomes) and four distance classes (nuclear genes) for Andean or mountain tapirs (*T. pinchaque*). (**A**) For overall mitogenomes; (**B**) For the nuclear *RAG 1-2* genes; (**C**) For the nuclear *IRBP* gene; and (**D**) For the nuclear *BRCA1* gene.

**Figure 9:**
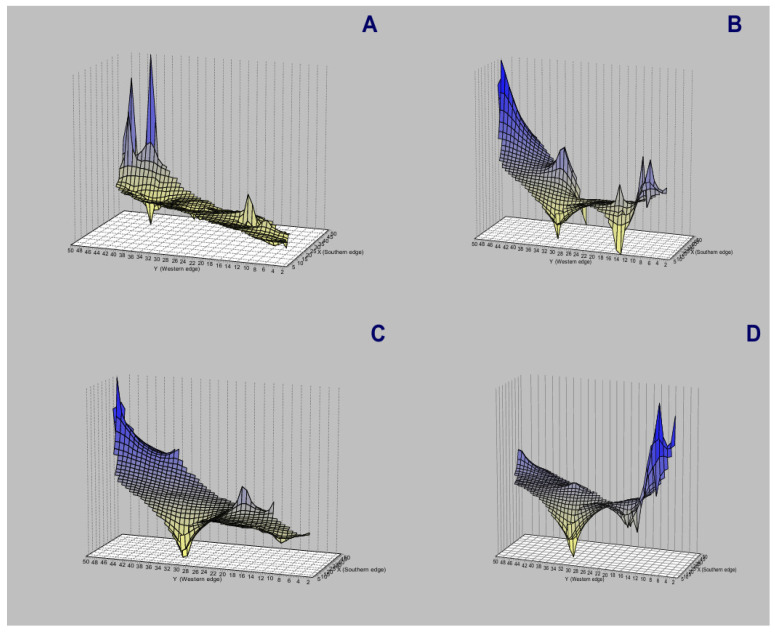
Genetic-landscape interpolation analysis (GLIA) for Andean or mountain tapirs (*T. pinchaque*) across Colombia and Ecuador. (**A**) For overall mitogenomes, the greatest peaks (the most differentiated) corresponded to the specimens sampled in the most northern area of the study (Los Nevados National Park in Caldas and Risaralda, Colombia); (**B**) For the nuclear *RAG 1-2* genes; (**C**) For the nuclear *IRBP* gene; and (**D**) For the nuclear *BRCA1* gene.

**Figure 10:**
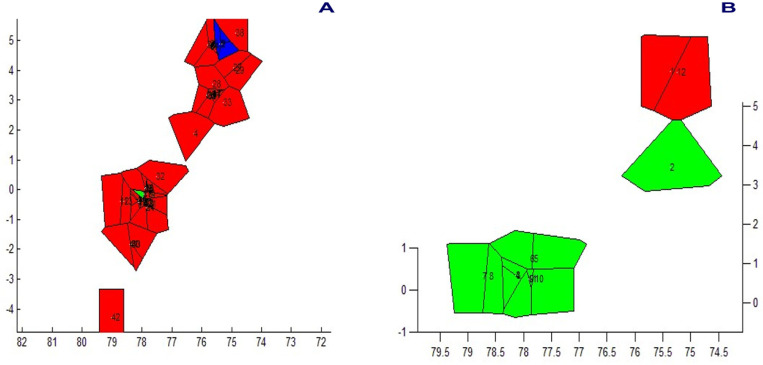
Different populations of Andean or mountain tapirs (*T. pinchaque*) detected by means of BAPS in Colombia and Ecuador using (**A**) overall mitogenomes; and (**B**) nuclear *RAG 1-2* genes.

The spatial genetic structure for complete mitogenomes is weak. The Mantel test yielded a slightly significant r value of 0.098 (*p* = 0.045), but the geographical distances only explained 0.96% of the genetic distances (Figure 7A). The overall correlogram (V = 0.00084, *p* = 0.075) did not reach statistical significance (Figure 8A), although two distance classes presented significant negative autocorrelations (2 DC: 29–61 km, *p* = 0.046; 5 DC: 272–327 km, *p* = 0.0019). Although, the spatial structure with mitogenomes was weak, the specimens of the most northern area sampled (Los Nevados NP in the Colombian Caldas and Risaralda Departments) were also the most differentiated when a GLIA was conducted (Figure 9A). The BAPS procedure (Figure 10A) detected the existence of three populations (log (ML) = −376.381) as the most probable situation: (1) the three specimens from Los Nevados NP in the Caldas Department, (2) one differentiated specimen from Chaco, Sarañan (Napo Province, Ecuador), and (3) all the remaining specimens. Thus, some specimens from the most northern distribution area of this species, in Colombia, appeared as the most differentiated.

For the *RAG1-RAG2* genes, the Mantel test was highly significant (r = 0.589, *p* = 0.0064), with the geographic distances explaining 34.7% of the genetic distances (Figure 7B). The overall correlogram was also highly significant (V = 0.00057; *p* = 0.0048) with the first DC positively significant (1 DC: 0–28 km, *p* = 0.011) and the fourth DC negatively significant (4DC: 304–409 km, *p* = 0.0056) (Figure 8B). The GLIA for the *RAG1-RAG2* genes clearly showed the differentiation of the most northern specimens in Colombia (Los Nevados NP) (Figure 9B). The BAPS procedure (Figure 10B) detected, as the most probable situation, the existence of two populations (log (ML) = −107.159). The specimens were either from Los Nevados NP (Risaralda, Colombia), or from throughout Colombia and Ecuador. Henceforth, again, the most northern distribution area (peripatric zone) contained the most differentiated specimens.

For the *IBRP* gene, the spatial genetic structure was also noteworthy. The Mantel test was highly significant (r = 0.494, *p* = 0.0103), with the geographic distances explaining 24.4% of the genetic distances (Figure 7C). The overall correlogram was also highly significant (V = 0.00091; *p* = 0.046) with the first DC positively significant (1 DC: 0–67 km, *p* = 0.013) and the fourth DC negatively significant (4DC: 297–409 km, *p* = 0.033) (Figure 8C). These results indicated that the specimens from geographically close areas were also those more genetically similar (in the first 0–67 km). Additionally, the most distant specimens (in this case, those from the Los Nevados NP in the Risaralda Department) were the most differentiated. The GLIA for the *IRBP* gene also showed the differentiation of the most northern specimens (Figure 9C). However, the BAPS procedure only detected one population, probably because of the reduced sample size for this gene.

For the *BRCA1* gene, the Mantel test did not detect any relationship between genetic distances and geographical distances (r = 0.213, *p* = 0.23). The spatial autocorrelation analysis did not reveal any spatial structure for this gene (V = 0.0079; *p* = 0.45) for the specimens of *T. pinchaque* studied. The GLIA for the *BCRA1* gene did not offer a clear view of the geographical differentiation of this species (Figure 9D). The BAPS procedure only detected one population probably because of the reduced sample size for this gene.

Henceforth, the nuclear *RAG1-RAG2* and *IRBP* genes showed a very marked spatial structure, whereas the mitogenomes yielded a weak spatial structure and the nuclear *BRCA1* gene did not offer any spatial structure. However, both types of molecular markers indicated that the most northern mountain tapirs sampled in the Nevados NP in Colombia have some genetic differentiation from the remaining specimens sampled in central-southern Colombia and throughout Ecuador.

### 3.4. Possible Demographic Changes in T. pinchaque

**Figure 11:**
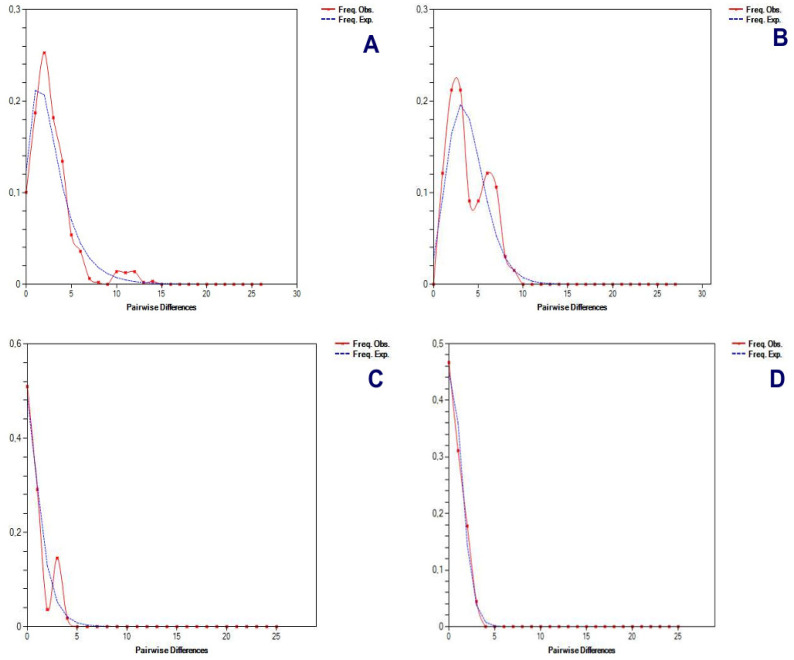
An analysis of significant mismatch distributions (pairwise sequence differences) for the Andean or mountain tapir (*T. pinchaque*) in Colombia and Ecuador. (**A**) For overall mitogenomes; (**B**) For the nuclear *RAG 1-2* genes; (**C**) For the nuclear *IRBP* gene; and (**D**) For the nuclear *BRCA1* gene.

**Figure 12:**
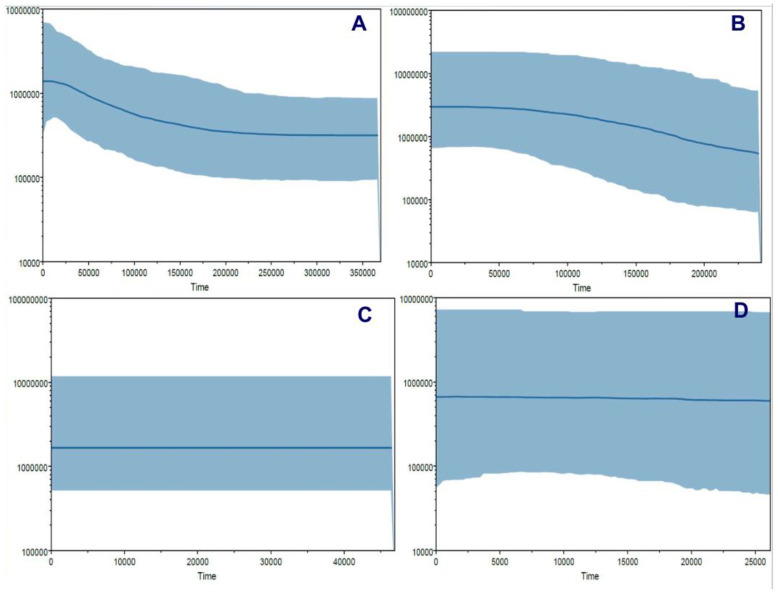
Bayesian skyline plot (BSP) analyses to determine the possible demographic changes across the natural history of Andean or mountain tapir (*T. pinchaque*) in Colombia and Ecuador. (**A**) For overall mitogenomes; (**B**) For the nuclear *RAG 1-2* genes; (**C**) For the nuclear *IRBP* gene; and (**D**) For the nuclear *BRCA1* gene.

**Figure 13:**
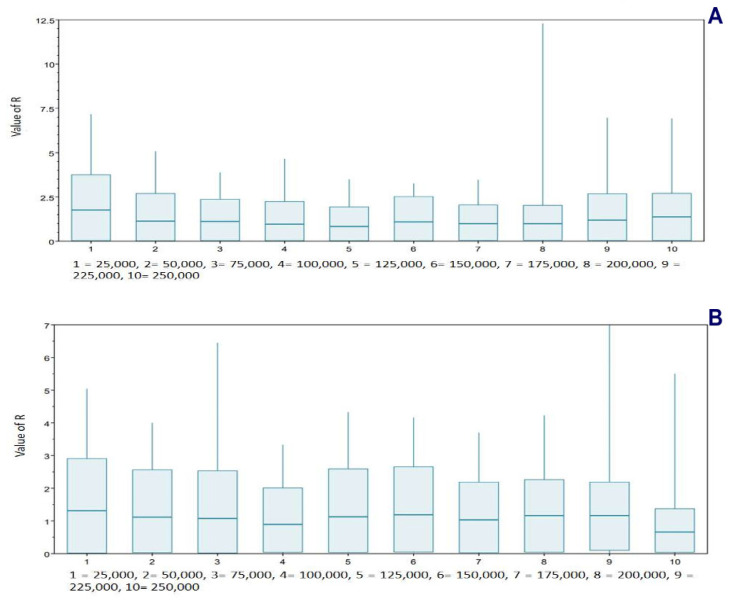
Birth–death model applied to the Andean or mountain tapir (*T. pinchaque*) sampled across Colombia and Ecuador to determine possible demographic changes. (**A**) For overall mitogenomes; (**B**) For the nuclear *RAG 1-2* genes.

Different procedures were applied to determine some possible demographic changes for *T. pinchaque* throughout its natural history. The mitogenome dataset showed strong evidence of population expansion for the mountain tapir. The mismatch distribution (Figure 11A) was significant (*rg* = 0.0223; *p* = 0.0334) and related to a demographic increase which began around 35,026 years ago. All the different statistics we used were significantly negative, which is related to a population expansion (D = −2.047, *p* = 0.0044; D* = −2.418, *p* = 0.0252; F* = −2.549, *p* = 0.0185; F_S_ = −11.690, *p* = 0.000001). The unique statistic which was not significant was the Ramos-Onsins and Rozas R2. The BSP procedure (Figure 12A) showed a slight and gradual population increase in the last 250,000 years ago. This increase intensified between 150,000 and 25,000 years ago. In the last 25,000 years, the female population was more constant. The birth–death model (Figure 13A) showed some increase since 250,000 to 200,000 years ago. Later, until around 75,000 years ago, the mountain tapir female population seemed to decrease to some degree. For 75,000 years ago, the population again increased with the highest increase occurring in the last 25,000 years.

The nuclear DNA markers also detected some population expansion for *T. pinchaque*. For the *RAG1-RAG2* genes, the mismatch distribution (Figure 11B) was significant (*rg* = 0.0450; *p* = 0.042) and related to a demographic increase which began around 11,151 years ago. Two statistics also correlated with population expansion (F_S_ = −9.708, *p* = 0.0001; R2 = 0.089, *p* = 0.0056). A slight and gradual population increase was detected from 200,000 to 50,000 years ago, and later, the population seems more or less stable with the BSP procedure (Figure 12B). The birth-death model (Figure 13A) showed a population increase in the last 50,000 years.

For the *IRBP* gene, the mismatch distribution (Figure 11C) was significant (*rg* = 0.0559; *p* = 0.0257) and related with a demographic increase which began around 2829 years ago. In addition, the Ramos-Onsins and Rozas R2 correlated with population expansion (R2 = 0.0685, *p* = 0.000001). The BSP procedure (Figure 12C) showed a constant population in the last 40,000 years. The sample size was too small to carry out the birth–death model.

For the *BRCA1* gene, the mismatch distribution (Figure 11D) was significant (*rg* = 0.0617; *p* = 0.0285) and related with a demographic increase which began around 19,203 years ago. Four statistics were also correlated with population expansion (D = −1.667, *p* = 0.0053; D* = −1.916, *p* = 0.006; F* = −1.897, *p* = 0.0359; F_S_ = −1.344, *p* = 0.0407). The BSP procedure (Figure 12D) showed a constant population in the last 25,000 years. Again, the sample size was too small to carry out the birth–death model.

Therefore, the overall results obtained revealed some evidence of population increase in *T. pinchaque* in the last phase of the Pleistocene.

## 4. Discussion

This is the second study on the molecular population genetics of *T. pinchaque*, but the first is to use nuclear gene sequences to obtain phylogenetic inferences of this species as well as for all the Neotropical tapir species.

### 4.1. Some Phylogenetic Inferences of the Neotropical Tapirs

The first phylogenetic result obtained re-affirmed those observed in other molecular studies [7,8,9,23,24,25,26]. *T. bairdii* corresponds to a branch which appeared first and is older than the branch that gave origins to *T. terrestris* and *T. pinchaque.* Thus, *T. terrestris* and *T. pinchaque* are sister species. Furthermore, it is observable that, within *T. bairdii,* there is a marked geographical differentiation; meanwhile, in *T. terrestris*, there are some groups related with specific geographical areas, but many groups presented specimens mixed from multiple origins [7,8,9,25,81]. In *T. pinchaque,* only a few specimens sampled in the northern periphery of its distribution in Colombia were slightly differentiated, but the remaining specimens from Colombia and Ecuador did not have any geographical structure [8]. Nuclear gene sequences agree quite well with the mitochondrial ones.

A second interesting phylogenetic result was related to temporal splits among the Neotropical tapir species. We estimated an average (mitogenome and the three nuclear sequence datasets) temporal split between the ancestor of *T. bairdii* and the ancestor of *T. terrestris* around 6.2 mya (final Miocene); meanwhile, the split of *T. terrestris* and *T. pinchaque* was estimated to have occurred around 1.9 mya (beginning of Pleistocene). The split of the *T. kabomani*’s lineage from other *T. terrestris*’ lineages occurred a little over 0.5 mya. Ashley and collaborators [23] analyzed the sequences of the mt *COII* gene and estimated the split between the ancestor of *T. bairdii* and the other two South American tapir species to have occurred around 20–18 mya. The split between the ancestors of *T. terrestris* and *T. pinchaque* was estimated to be around 2.7–2.5 mya; meanwhile, using the mt *12S rRNA* gene, these split events are estimated to have occurred around 16.5–15 mya and 1.6–1.5 mya, respectively [24]. Ruiz-García and collaborators [25], using the mt *Cyt-b* gene, determined that the ancestor of *T. bairdii* diverged around 10.9 mya (95% HPD: 6.3–16.3 mya) from the *T. terrestris*–*T. pinchaque* clade and the ancestors of *T. terrestris* and *T. pinchaque* diverged around 3.8 mya (95% HPD: 3.1–4.7 mya). In fact, using a median joining network, the most frequent *T. terrestris* haplotypes diverged from the main *T. pinchaque*’s haplotype around 1.55 ± 0.32 mya. Later, another study of the same team, analyzing 15 mitochondrial genes (two rRNA and 13 protein codifying genes), determined a temporal split of the ancestor of *T. bairdii* relative to the ancestor of the other South American tapirs to have occurred around 8.1 MYA (95% HPD: 10.5–4.63 mya); meanwhile, the split between the ancestors of *T. terrestris* and *T. pinchaque* was estimated to have occurred around 3.7 mya (95% HPD: 7.42–3.27 mya). Additionally, another study determined that the evolutionary rates of chromosomal rearrangement were extremely low in all ceratomorph perissodactyl lineages (0.3 rearrangements per million years, R/my), with fissions predominating among the chromosomal rearrangements that were identified. The only exceptions were two tapir species (*T. indicus* and *T. pinchaque*), in which Robertsonian fusions occur at a slightly elevated rate (0.62–0.77 R/my) compared to other lineages [82]. *T. pinchaque* showed three rearrangements in reference to *T. terrestris*, which offered a temporal divergence between the branches of *T. terrestris* and *T. pinchaque* around 4.83–3.89 mya.

One unique work [4] disagrees with all these molecular and chromosomal temporal estimates. In that study [4], a divergence time of around 0.1–0.3 mya was found between *T. terrestris* and *T. pinchaque*. These authors applied a constriction for the mitochondrial haplotype diversification in *T. terrestris* of 0.13 ± 0.1 mya because they affirmed that the oldest fossil records of *T. terrestris* date back to the beginning of the fourth Pleistocene glaciation (0.13 mya). However, as we will comment below, the existence of *T. terrestris* is considerably older than 0.13 mya. This uncorrected paleontological constriction helps to explain the difference in temporal divergence between South American tapir species noted by Cozzuol and collaborators [4] relative to other studies, as well as the appearance of *T. kabomani* as a “full” species (Table 2).

Our split estimates, including nuclear sequences, were somewhat lower than those obtained with mitochondrial genes especially the split between *T. bairdii* and *T. terrestris–T. pinchaque*, probably because the substitution rates of nuclear genes are considerably lower than the mitochondrial substitution rates. The split between *T. terrestris* and *T. pinchaque* we reported here is inside the range of the temporal splits obtained in other works.

Based on our analysis, the average temporal genetic diversification within *T. terrestris* and *T. bairdii* occurred during the Early Pleistocene around 1.7 and 1.2 mya, respectively. If the sample of *T. bairdii* would have had a larger magnitude—on par with that of *T. terrestris*—the temporal genetic diversification for *T. bairdii* would probably be even greater than that determined for *T. terrestris*. In contrast, the average temporal genetic diversification within *T. pinchaque* is clearly much more recent. It occurred around 0.5–0.2 mya in the last phase of the Pleistocene. This value was lower than other *T. pinchaque*’s temporal mitochondrial diversification estimates: around 2.1 mya (95% HPD: 1–3.3 mya), and 2.9 mya (95% HPD: 6.09–2.68 mya), respectively [8,25]. Again, the within genetic diversification, including nuclear gene sequences, diminished the times of diversification in the different tapir species studied. However, these last two research studies, along with the current work, provide evidence that *T. pinchaque* is the youngest Neotropical tapir species, which disagrees radically with the assumptions previously made by some zoologists and paleontologists. In fact, there is not currently any fossil related to *T. pinchaque*, which is probably related to the fact that it is the youngest tapir species as well as that the Andean highlands are not the better areas to obtain certain kinds of fossils. For example, some authors used morphological characters and determined that the lineage which gave rise to *T. pinchaque* was the oldest of the current Neotropical tapir species because it is the least specialized tapir [18,20,83]. The conclusion by Hershkovitz [18] was based on the close similarity of cranial contours of *T. pinchaque* to some fossil tapir skulls which have a less specialized proboscis than current tapir species. This author commented on the close resemblance of *T. pinchaque* and the early tapirid *Protapirus* and tapiroid *Heptodon*. Also, a modern study associated *T. pinchaque* with some old fossil species [84]. This study showed a considerable overlap between *Nexuotapirus marlandensis* and *T. lundeliusi*, with *T. johnsoni* and *T. pinchaque* were near this overlap. It was commented that these four species exhibit similar cranial shape, including a dorso-ventrally flattened skull, elongated rostrum, and sagittal crest extending straight to the nasals. Furthermore, *T. pinchaque* exhibits elongated nasals, a condition referred to as primitive inside *Tapirus* [85].

However, all these morphological and morphometric skull analyses disagree with all the molecular studies which showed *T. pinchaque* as the youngest Neotropical tapir species (both mitochondrial and nuclear genes as shown in the current work). This is the problem of using craniometrical and teeth data when dealing with tapirs because nobody knows the degree of phenotypic plasticity of these characters and their genetic basis. The cranial similarities shared by those tapir species (*Nexuotapirus marlandensis*, *T. lundeliusi*, *T. johnsoni*, and *T. pinchaque*) were probably homoplasy which shows the debility of these craniometric studies with tapirs for obtaining strong phylogenetic inferences. Additionally, molecular data neither support the paleontological hypothesis of some authors that *T. pinchaque* and *T. terrestris* correspond to well differentiated lineages of tapirs [18,83]. Our mitogenomic and nuclear results are more in line with the paleontological view of Hulbert and colleagues [85,86], because they consider that *T. pinchaque* and *T. terrestris* form a monophyletic group.

Another interesting fact is that the small genetic distances found for diverse molecular markers between *T. pinchaque* and *T. terrestris* have been used by some authors to speculate about the possibility that these tapirs are not different species [6,7,8]. For instance, Voss and collaborators [6] commented that striking ecogeographic variation is well known among widespread large mammals [87,88], and it is not impossible for *T. pinchaque* to be a high-altitude ecomorph or subspecies of *T. terrestris*. Indeed, we believe that the ancestor of *T. pinchaque* derived from an ancestral form of *T. terrestris*. However, we also believe that *T. pinchaque* is a full species despite the small molecular differences with *T. terrestris*. *T. pinchaque*, *T. terrestris*, and *T. bairdii* differ in the number of diploid chromosomes and FN (*T. pinchaque* 2N = 76, FN = 80; *T. terrestris* 2N = 80, FN = 80–92; *T. bairdii* 2N = 80, FN = 94) [89,90]. The autosome chromosomes of *T. pinchaque* consist of six metacentrics/submetacentrics and 68 acrocentrics/telocentrics. The Y chromosome is a small acrocentric and the X is a large submetacentric. In G- and C-banded preparations, the X chromosomes of *T. bairdii,* and *T. terrestris* were identical, whereas the X chromosomes of *T. pinchaque* differed by a heterochromatic addition/deletion. The Y chromosome was a medium-sized to small acrocentric in *T. bairdii, T. indicus,* and *T. pinchaque,* but it was extremely small in *T. terrestris*. Furthermore, G-banded karyotypes indicated that a heterochromatic addition/deletion distinguished chromosome 3 of *T. pinchaque* from the remaining species of *Tapirus*. Additionally, there were at least 13 conserved autosomes between the karyotypes of *T. bairdii* and *T. terrestris,* and at least 15 were conserved between *T. bairdii* and *T. pinchaque.* Thus, from a chromosomal point of view, and probably from a reproductive (Biological Species Concept, BSC) [91,92], and from an ecological perspective, *T. pinchaque* is a full species.

### 4.2. The Inexistence of T. kabomani as a Full Species

Cozzuol and collaborators [4] claimed to describe a new species of tapir from the Amazon (*T. kabomani*). They affirmed that they discovered the first new species of Perissodactyla in more than 100 years, from both morphological and molecular characters. They said that this new tapir species was shorter in stature than *T. terrestris* and had a distinctive and different skull morphology compared to that of *T. terrestris.* For example, it had a lower sagittal crest, broad and inflated frontal bones posterior to the nasal bones that extended up to the frontal-parietal suture. However, the same authors considered that the nasals of *T. kabomani* were like those of *T. terrestris* in shape and did not project upward, as in *T. bairdii* and *T. pinchaque*. They also claimed that within the phylogenetic tree they constructed, *T. kabomani* was basal to the clade formed by *T. terrestris* and *T. pinchaque.* This phylogenetic tree was based on mt *Cyt-b* gene sequences of 52 *T. terrestris*, 5 *T. pinchaque*, 4 *T. kabomani*, and 3 *T. bairdii*. Many of these specimens were previously studied in other work [81]. Another phylogenetic tree based on the analysis of three mt genes (*Cyt-b*; *COI*; *COII*) from 6 *T. terrestris*, 1 *T. pinchaque*, 3 *T. kabomani*, and 1 *T. bairdii* was used by Cozzuol and collaborators [4] to vindicate *T. kabomani* as a full new species. Furthermore, it was claimed that *T. kabomani* lives in habitats which are mosaics of forest and patches of open savanna, probably, Holocene relicts of Cerrado. They also concluded that where only forest or open areas are dominant, *T. kabomani* seems to be rare or absent. Moreover, they agree with the local peoples about the existence of two different forms of tapirs in the southern Brazilian Amazon.

Nevertheless, an accurate analysis of these morphological, molecular, ecological, and ethnozoological considerations does not support *T. kabomani* as a full tapir species [6,7,8,9], and the current work.

From a morphological perspective, there are many problems with *T. kabomani*. First, it was commented that the ontogenetic variation of the skull of *T. terrestris* is potentially problematic [6]. Cozzuol and collaborators [4] claimed that specimens with M1 erupted are already sexually mature and the skull and size subsequently change little or not at all. However, it has been demonstrated that the 1st upper molar (M1) of tapirs erupts while the deciduous premolar dentition (dP2–dP4) is still in place [6]. In fact, specimens that retain dP2–dP4 are considered juveniles by most tapir taxonomists [85,86,93], who consider only specimens with erupted P4/p4 to be fully grown. For instance, it was considered that *T. pinchaque* reaches a definitive adult size (in terms of skull length) by the time M2 appears [18]. Thus, the criteria used by Cozzuol and collaborators [4] seems highly subjective.

Second, Ruiz-García and collaborators ([7] and unpublished) analyzed the craniometrics of several *T. terrestris* populations throughout different areas of Colombia, Ecuador, Peru, Brazil, Bolivia, Paraguay, and Argentina (210 skulls of *T. terrestris*, 6 *T. pinchaque*, 1 *T. bairdii*). Most of the *T. terrestris* populations showed strong significant statistical differences regarding the two areas of the skull where Cozzuol and collaborators [4] found the greater divergence of *T. kabomani* regarding *T. terrestris* (the position of the frontal-parietal suture in relation to the beginning of the sagittal crest and frontal broad and more inflated behind the nasals in *T. kabomani*). Significant differences of the skulls we studied within diverse *T. terrestris* populations (1—Napo River area in northern Peruvian Amazon; 2—Yavarí River area in Brazil and Peru; 3—northern Colombia; 4—Colombian Eastern Llanos; 5—Ecuadorian Amazon; 6—Bolivian Amazon; 7—Paraguay and Argentinian Chaco; and 8—Argentinian Misiones province) were related to different ontogenetic patterns within each population as well as to the age composition of each population. However, we don’t claim that each of these populations was a different tapir species. In fact, we detected a strong and significant correlation between an index to measure the size of the sagittal crest and the number of definitive erupted molars and premolars. The specimens with the small sagittal crest index (as is expected in *T. kabomani*) also had the lowest number of definitive erupted molars and premolars. That is, they were the youngest adults studied. Curiously, the two alleged Brazilian *T. kabomani* (holotype, UFMG 3177, and the paratype, UFMG 3176) studied by Cozzuol and collaborators [4] used from a molecular perspective, were young male specimens. Moreover, we detected that the temporal ontogenic trajectory strongly varies across different specimens from different populations of *T. terrestris* (within and between populations). For instance, we observed a very large body specimen of *T. terrestris* that had an erupted M1 but also, the color markings and small sagittal crest typical of infants in Bolivia (with typical mt haplotype of *T. terrestris*). We also observed small-sized *T. terrestris* specimens in the Colombian and Ecuadorian Amazon (similar phenotype of *T. kabomani*). Each had very dark colors, an erupted M3 (old adult specimens) and typical mt haplotypes of *T. terrestris*. Furthermore, we even analyzed a “typical” morphological *T. terrestris* specimen from Tena (upper Napo River in Ecuador) that had a considerable size, light brown color, and a well-developed sagittal crest, and a mt haplotype of *T. kabomani*. Thus, we did not detect a correlation between the morphotype proposed for *T. kabomani* and the *T. kabomani* mt haplotypes. Additionally, another work [94] completed the importance of the ontogenetic process in the development of the skull of *T. terrestris*. They conducted univariate, multivariate and allometric analyses of 32 skull variables of 54 specimens sampled from a population in the Loreto Department, in the Peruvian Amazon. Significant skull shape variation was detected among ontogenetic classes. Young individuals (class I, young juvenile; molar 1 fully erupted, molars 2 and 3 not erupted; individuals under one year of age) showed higher variation than subadults (class II, subadult to young adult; molars 1 and 2 erupted, molar 3 not erupted; individuals between 1 and 6 years), and adults (class III, adult to old adult; all dental elements fully erupted; individuals older than 6 years), without evidence of sexual dimorphism. But the variation in classes II and III were also significant. This does not support previous affirmations [4,95] that the skull structure of *T. terrestris* is largely conserved throughout the postnatal ontogeny (after the eruption of molar 1). Moreover, it was shown [94] that allometric analyses indicated a tendency of unproportioned cranial growth. They observed allometric trends, which were isometric in four (12.5%) of the 32 skull measurements. Eleven (34.3%) showed a positive allometry and 16 (53.1%) showed a negative allometry. The changes were primarily related to relative differences in the growth of the facial skeleton compared to the braincase, showing that *T. terrestris* follow the generalized mammalian pattern of greater relative growth of the face compared to the braincase as the animals mature [96]. This could explain why in the older specimens of *T. terrestris*, there is a shorter length of the frontal area and a less inflated structure posterior to the nasals in reference to the alleged *T. kabomani* (always integrated by young adults). A previous work [95] did not detect this facial growth in their study, probably because it was obscured by regional variation throughout the geographical distribution of *T. terrestris*.

Because the samples studied in the mentioned study [94] come from the same population living under the same ecological condition, this eliminates the effect of confounding variables related to habitat. This work also shows that the pattern of ontogenetic variation in the skull of *T. terrestris* is extremely heterogeneous in specimens within the same population. The ontogenetic changes reported can be associated with the skull development of tapirs and their ability to select specific food materials [97]. Their results suggest that neurocranium and splanchnocranium begin to differentiate slightly between subadults (class II) and adults (class III) depending on their feeding. Availability and quality of food resources across different ages are likely to affect the morphology of the skull, both because of a general influence on growth rates and locally at the level of skull structure [98]. This shows the extreme plasticity of the tapir skulls depending on their feeding. In fact, it was shown that the discovery of 75 individuals of *T. polkensis* in the Gray Fossil Site in Eastern Tennessee indicated a unique species [86]. However, it had considerable intraspecific variation in the development of the sagittal crest, outlined shape of the nasals and the number and relative strength of lingual cusps on P1. It was concluded that if these fossil remains had been found in diverse geographical areas, they would have been considered different species. This extreme phenotype plasticity was misunderstood by Cozzuol and collaborators [4]. Therefore, the existence of morphological and morphometric skull variability in the lowland tapirs does not necessarily mean the real existence of different tapir species.

Third, Cozzuol and collaborators [4] described *T. kabomani* as having more inflated frontals than *T. terrestris*, and they scored this last species as having uninflated or weakly inflated frontals. However, as it was commented [6], Holanda and Ferrero [83], in an article on South American tapir systematics, described and scored *T. terrestris* as having strongly inflated frontals. This suggests that character scoring based on subjective criteria should not be a part of determining tapir taxonomy.

Fourth, Cozzuol and collaborators [4] carried out a skull cladistics analysis of different fossils and current tapir taxa. They determined that the grouping of *T. indicus* with *T. bairdii* and several North American fossil species is the major difference between phylogenetic results of morphological and DNA characters (molecular data always show the monophyly of the Neotropical tapirs in reference to the Asian tapir, *T. indicus*). They claimed that the reason for this is not clear. However, for us, the reason is very clear. Some, or many, of the skull characters they used in the skull morphometric cladistics analysis are not useful in phylogenetics. For example, subjective criteria were used in the selection of characters. Additionally, this research understands neither the degree of phenotypic plasticity of these characters nor their genetic basis.

Additionally, another study [84], using geometrical morphometrics, showed that *T. kabomani* is within the skull variation of *T. terrestris*. This study proposed landmarks for the study of tapir cranial shape through 2D geometric morphometrics. It included 20 in lateral cranial view, 14 in dorsal cranial view, and 21 in ventral cranial view, followed by PCA multivariate statistical analysis that ordinated specimens from each of the three data groups along the major axis of shape variation. Lateral and dorsal view landmarks proved to be the most diagnostic for the species studied. The tapir species they analyzed (both fossil and living species) did not significantly differ ventrally. The results of their PCA diagram of 20 lateral view landmarks (PC1 [25% of total variation] versus PC2 [15% of total variation]), for extant and extinct species of tapiroid species, clearly showed that *T. kabomani* and the extinct *T. mesopotamicus* were overlapped with *T. terrestris*. However, *T. pinchaque* and *T. bairdii* were not overlapped with *T. terrestris*. This was the most diagnostic analysis in that analysis [84]. A PCA matrix for 14 dorsal view landmarks of PC1 versus PC3 (74% of the total variance between species), for extant and extinct species of Tapiridae species, showed even more evidence that *T. kabomani* is inside the variation of *T. terrestris*. Therefore, lateral view landmarks proved to be the most diagnostic ones for the data analyzed, followed by dorsal view landmarks and both identified *T. kabomani* as an extension of *T. terrestris*, while *T. pinchaque* and *T. bairdii* were clearly differentiable from *T. terrestris*.

The molecular results also do not support the existence of *T. kabomani* as a full species as we showed in the current work. First, Voss and collaborators [6] commented that the nodal support for *T. kabomani* was weak in the mt*Cyt-b* phylogenetic tree of Cozzuol and collaborators [4] and only moderate likelihood support was recovered from their three concatenated-gene data set (mt *COI-COII-Cyt-b*). Second, it was also claimed that the mean distance between mt *Cyt-b* sequences attributed to *T. kabomani* and those from *T. terrestris* was only 1.3%, which is well within the range of sequence divergence values routinely reported among conspecific mammalian haplotypes [99]. Similarly, another study [7] showed that the Kimura 2P genetic distances between *T. terrestris* and *T. kabomani* for the three mt genes (*COI-COII-Cyt-b*) were low (average for the three genes: 1.2%), typical of populations of the same species, and smaller than the genetic distances between *T. terrestris* and *T. pinchaque* (*Cyt-b*: 1.8% ± 0.3% vs. 2.4% ± 0.3%; *COI*: 0.5% ± 0.2% vs. 1.0% ± 0.4%; *COII*: 1.3% ± 0.3% vs. 1.7% ± 0.4%, respectively). Additionally, the same study [7] showed an MJN procedure for the three concatenated mt genes (*COI-COII-Cyt-b*) that indicated that the haplogroups of *T. bairdii*, *T. terrestris,* and *T. pinchaque* were well defined. However, the *T. kabomani* haplotypes were an extension from those of *T. terrestris*. Furthermore, that study also analyzed possible demographic changes in *T. terrestris* and *T. kabomani*. The mismatch distribution and diverse demographic tests showed evidence of similar population expansion in both taxa. Thus, *T. kabomani* was a dynamic demographic extension of *T. terrestris* [7]. Third, Ruiz-García and collaborators [9] showed a maximum likelihood tree for the three quoted mt genes concatenated (*COI-COII-Cyt-b*), representing all Neotropical tapir species and it was based on larger sample size relative to the tree showed by Cozzuol and collaborators [4]. There was no evidence that *T. kabomani* should be a full species, rather it was only a haplogroup within *T. terrestris*. Clearly, to augment the number of *T. pinchaque* individuals sequenced with reference to Cozzuol and collaborators [4], *T. pinchaque* was more differentiated from *T. terrestris* than *T. kabomani*. The inaccurate result obtained in that study was probably related to the fact that these authors only analyzed five samples of *T. pinchaque* at the mt*Cyt-b* gene and only one sample at the set of *Cyt-b + COI + COII* genes. Indeed, they only analyzed three samples at the mt*Cyt-b* gene, because two *T. pinchaque* samples were repeated due to that the two samples that Ruiz-García donated to the Cozzuol’s team were also donated by another scientist. The extremely small sample size of *T. pinchaque* used by Cozzuol and collaborators [4] probably did not represent an accurate mitochondrial genetic diversity of *T. pinchaque*. Furthermore, as the genetic differences between *T. terrestris* and *T. pinchaque* are very small, by chance the low mitochondrial genetic diversity of the very small sample size of *T. pinchaque* used by Cozzuol’s team was nested within the genetic diversity of *T. terrestris*. Nevertheless, once the sample size of *T. pinchaque* was enlarged, this phenomenon was eliminated and *T. kabomani* disappeared as a full species [8,9].

The four new data sets (mitogenomes, and nuclear *RAG1-RAG2*, *IRBP*, and *BRCA1* genes) herein clearly showed and ratified that *T. kabomani* is a group within *T. terrestris*. For instance, our mitogenome data set of 156 tapirs clearly refutes that *T. kabomani* is a new species. It should be noted that the molecular result of Cozzuol and collaborators [4] is based on one unique mt gene (*Cyt-b*), and gene trees do not necessarily represent species trees. The fact that Cozzuol and collaborators [4] showed several autapomorphic molecular characters to define *T. kabomani* as a differentiable clade from other *T. terrestris*’ clades, does not mean that *T. kabomani* is a full species. We also detected multiple molecular autapomorphic molecular characters for the clade of *T. kabomani* in the new marker sets that we present here, but always in a context of low, or very, low genetic distances with reference to other groups of *T. terrestris* and always in a context within *T. terrestris*.

Evidence provided by chromosomal studies [100] also refutes the existence of *T. kabonmani* as a full species. That study analyzed the karyotypes of seven *T. terrestris* from the Wroclaw Zoo (Poland). Each specimen’s phenotype differed from that of the others. For example, some were small and very black, like the alleged *T. kabomani*. The sampled individuals even differed in sexual behavior as well. However, despite these considerable morphological and behavior differences among these specimens, each possessed karyograms with an equal diploid chromosome number (2n = 80). Homologous chromosomes did not differ between each other with quantity, size, centromere location, length of arms, G bands and all were classified as karyograms of *T. terrestris*. The X chromosomes as well as the first pair of chromosomes (both metacentric), were the largest among all analyzed, respectively. All the remaining 38 pairs of chromosomes were acrocentric with Y chromosome as the smallest one. Henceforth, despite conspicuous morphological differences, all the specimens were classified as *T. terrestris*, showing that many morphological characters in *T. terrestris* have a great degree of phenotypic plasticity, and are therefore not useful as phylogenetical characters.

The karyotypes of *T. terrestris* and other tapir species (like *T. bairdii*) can be already distinguished based on classical cytogenetic examination, using only the shape of large chromosomes and position of centromeres in them. It was demonstrated the differences in the shape of a few large chromosomes, caused by different positions of centromeres, presence of shorter arms, and their size: chromosome 1 of *T. terrestris* is metacentric, while in *T. bairdii* it is submetacentric, and chromosome 8 in *T. terrestris* is acrocentric, while in *T. bairdii* it is metacentric [90]. Additionally, in the same work [90], based on G banding in karyotype of *T. bairdii*, demonstrated large changes in type of insertion/deletion in chromosome 2, which can be used in the differentiation from other tapir species. Henceforth, the different tapir species can be differentiated by their karyotypes, but “typical” *T. terrestris* morphotypes and tapirs morphologically like the alleged *T. kabomani* could be not karyotypically differentiated.

Nonetheless, some chromosome variability has been demonstrated within *T. terrestris*, but neither associated with the characters of the alleged *T. kabomani* nor in the geographical area where it could live. A major part of the *T. terrestris* analyzed (included those with appearance of *T. kabomani*) showed chromosome 1 and X as being metacentric, and all others as being acrocentric [101]. However, some specimens from northern Colombia of the alleged *T. terrestris colombianus*—the largest form of *T. terrestris*, have a karyotype that contains metacentric chromosomes (1 and X), and 6 submetacentric pairs [102]. The remaining chromosomes are acrocentric. Also, another work [103] reported the karyotypes from some possible *T. terrestris colombianus* with FN = 92 (number of autosomal arms), when the typical FN for the remaining forms of *T. terrestris* is FN = 80. These authors described chromosome 1 as metacentric, 6 autosomes as subtelocentric, and 32 consecutive autosomes as acrocentric. Therefore, the specimens of *T. terrestris,* which showed chromosome polymorphism in reference to the most frequent karyotype of *T. terrestris,* was the largest form of this species, *T. t. colombianus*, not the alleged *T. kabomani*. It had a very large and high sagittal crest, and a dark brown pelage color.

Another alleged argument of Cozzuol and collaborators [4,5] was ecological. They affirmed that *T. kabomani* inhabits areas which have both forest and open areas. In other words, they concluded that where only forest or open areas are dominant, *T. kabomani* seems to be rare or absent. The two Colombian *T. kabomani* provided by Ruiz-García to the Cozzuol’s team (one of them was amplified for the overall mitogenome and for the three nuclear marker sets used; the other sample did not work for these new molecular markers), as well as the other five *T. kabomani* specimens that we detected in previous works [7,8,9] and used in this work come from primary Neotropical rain forests (not altered). Henceforth, the claim that *T. kabomani* is only present where mosaics of forest and patches of open savanna are present, is also incorrect.

We do agree with Cozzuol and collaborators [4] on the fact that the haplogroup of the alleged *T. kabomani* within *T. terrestris* likely originated during environmental conditions of Refugia (Refugia Hypothesis). This hypothesis claims that alternative dry/humid cycles in the Amazon basin, as the result of Milankovitch cycles, created different isolated refuges during the dry periods of the Pleistocene, due to forest fragmentation [104,105,106,107,108]. During the dry periods, rainforest communities split into isolated refuges separated by savannah or arid pampas. This hypothesis could explain the appearances of the different haplogroups detected within *T. terrestris*. During each dry period, the haplogroups (one of them, *T. kabomani*) were formed, but later during the humid periods some of these *T. terrestris*’s haplogroups migrated allowing the diversification of them in different sympatric geographic areas. We found the alleged *T. kabomani* sympatrically living with other haplogroups of *T. terrestris*. Some Pleistocene refuges could have played an important role in the origins of these *T. terrestris*’s haplogroups. The *T. kabomani*’s haplogroup could have radiated from the Napo refuge (Northwestern Amazon), while other *T. terrestris*’s haplogroups could have radiated from the Napo and Inambari refuges (Southwestern Amazon). The haplogroup of the southern *T. terrestris* could have expanded from the Inambari or from the Rondonia refuges (between the Madeira and the Tapajos rivers), whilst the most northern *T. terrestris*’s haplogroup could have originated from the Bolivar refuge within the Guiana area.

Finally, Cozzuol and collaborators [4,5] gave total credit to the local indigenous people for their knowledge. These indigenous peoples recognize two different forms of tapirs in the southern Brazilian Amazon. Traditional indigenous knowledge can be helpful in our understanding of taxonomic groups, but not always, and not in this case. For example, in different areas of the Colombian and Peruvian Amazon, some Indian tribes have differentiated up to four different forms of tapirs even where only one species (*T. terrestris*) is present. In another example, some Amazon indigenous tribes have identified up to six different forms of river dolphins although only two species (*Inia geoffrensis* and *Sotalia fluviatilis*) (Ruiz-García, unpublished observations) are present. In each of these cases, determination of each “species” was based on size and color. Many of these differences are related to age classes.

Finally, a commentary on the species concept. Cozzuol and collaborators [4,5] never defined which species concept they used to claim *T. kabomani* as a new species. There are many definitions of species [109]. Like the most conspicuous results from Cozzuol and collaborators [4] was that *T. kabomani* formed a “significant cluster” apart from *T. terrestris* in their molecular phylogenetic trees and *T. kabomani* has a “supposed” distinguishable morphology and morphometrics from *T. terrestris*, thus, they would have implicitly used the Phylogenetic Species Concept 1 (PSC1) [110] and the Genotypic Cluster Species Concept (GCSC) [111]. PSC1 defined a species as an irreducible cluster of specimens that is significantly distinct from other similar clusters and with a clear pattern of ancestry and descent. Here, we showed that *T. kabomani* does not meet this definition. On the other hand, GCSC defined a species as a morphological or genetical distinguishable cluster of individuals that has no intermediates when it is in contact with other similar clusters. For instance, from a morphological perspective, we showed as the craniometric characters employed by Cozzuol and collaborators [4,5] were influenced by the age of the specimens used and that tapirs “morphologically” like *T. kabomani* have DNA “typically” from *T. terrestris* and vice versa. Therefore, GCSC does not differentiate *T. kabomani* as a full species from *T. terrestris*.

Identically, other definitions of species do not probably differentiate both tapir forms. The fact, that specimens with the “phenotype” of *T. kabomani* had “typical” DNA from *T. terrestris* and had not chromosome differences with this last taxon, probably indicates that there is reproductive cohesion between both forms. Henceforth, the definitions of BSC (probably the most important one), Recognition Species Concept (RSC; a species is the most inclusive population of biparental individuals sharing a common fertilization system) [112], or Evolutionary Species Concept (EvSC; a species is a single lineage of ancestral descendant populations maintaining its identity from other lineages having its own evolutionary and demographic tendencies) [113], also do not differentiate *T. kabomani* from *T. terrestris*. Even, the Ecological Species Concept (EcSC; a species is a linage which occupies an adaptative zone different from that of other similar lineages which separately evolve outside from this adaptative area) [114] neither differentiate *T. kabomani* from *T. terrestris* because we previously commented that *T. kabomani* lives together with *T. terrestris* in the same types of Amazonian forests.

Thus, independently of the species definition used, *T. kabomani* is very unlikely to be a different species from *T. terrestris*.

### 4.3. Genetic Diversity and Spatial Structure in T. pinchaque

This work and another [8] showed that *T. pinchaque* yielded the lowest levels of genetic diversity for mitogenomes compared to *T. terrestris* and *T. bairdii*. In the current work, *T. pinchaque* showed the lowest values of genetic diversity both for mitogenomes and for the three nuclear DNA sequence data set. This could be correlated with three facts. It is the youngest Neotropical tapir species; it originated from a strong founder effect from some ancestral *T. terrestris* population; and it is the Neotropical tapir species with the smallest geographical distribution area. For example, population densities for *T. pinchaque* were estimated at 1 individual/587 ha in Sangay National Park, Ecuador [115], 1 individual/400 ha in Ucumari Regional Park, Colombia [116], and 1 individual/551 ha in Los Nevados NP, Colombia [117]. Therefore, *T. pinchaque* has the lowest population density of all Neotropical tapirs [117]. These historical constrictions could negatively affect the genetic diversity and thus the survival of this emblematic Andean species. We must have in mind that *T. pinchaque*, one of the rarest large mammals in the world, is listed under Appendix I of the Convention on International Trade in Endangered Species of Fauna and Flora (CITES) and is considered “Endangered” by the International Union for Conservation of Nature and Natural Resources (UICN). The number of specimens of *T. pinchaque* in the Colombian Andes has been estimated at 2500 animals in 35 forest patches, and of them, only five or six are large enough to maintain the minimal number of animals for a viable population in the short term [13].

Another interesting feature, for the three tapir species, is that mitogenomes always showed levels of genetic diversity higher than did sequenced nuclear genes. This means that, a priori, analyses using mitochondrial markers could detect more diversity, and have potentially more power for discriminating groups or structure within species, or very, related species.

The differentiated results of the spatial structure analyses varied with the molecular markers used. Although, the spatial structure with mitogenomes is weak for *T. pinchaque*, the specimens of the most northern area sampled in the Colombian Central Cordillera (Los Nevados NP in the Caldas and Risaralda Departments) were also the most differentiated when the GLIA and BAPS procedures were conducted. Differently, the sets of *RAG1-RAG*2 and *IRBP* detected strong spatial structure for the specimens from Los Nevados NP. This is a peripatric area from the most northern distribution of this species in the Colombian Central Cordillera. In contrast, *BRCA1* did not detect any relevant spatial structure for *T. pinchaque*. Probably, this little northern *T. pinchaque* population (Los Nevados NP) is in the periphery of the geographical distribution of this species in the Colombian Central Cordillera. Consequently, it has undergone more intense genetic drift/founder effects (detected by mitogenomes) and/or it has been subjected to differential selective pressures which are different from those that affected the main distribution area of the species (detected by *RAG1-RAG2*, and *IRBP*). This could explain why specimens from Los Nevados NP presented some molecular differences relative to the remaining specimens studied. Unfortunately, this small *T. pinchaque* population is in a critical situation. For example, potato farmers, living within Los Nevados NP, kill this species when it raids their crops [118].

What is clear is that there are non-significant, or very limited, genetic differences among mountain tapir populations (excluding Los Nevados NP). This means that historical gene flow has been sufficient to avoid genetic differentiation, although the Andean habitat is currently extremely fragmented. Fragmentation might be very recent, thus preventing genetic differentiation of the *T. pinchaque* populations. Another possibility might be high current gene flow. This has been demonstrated for *T. terrestris* [119]. For *T. pinchaque*, probably, the capacity of gene flow is lower than for *T. terrestris*, although the degree of movement could be relatively important. With global position system collars, a male moved 94.1 km over a 6-month period, and two other specimens each traveled 91.4 km over 6 months [120]. The non-existence of spatial genetic structure is an interesting fact from a conservation perspective because there should not be genetic consequences if animals are translocated (at least, excluding the specimens from Los Nevados NP).

### 4.4. Demographic Changes

Analysis of mitogenomes for *T. pinchaque* indicates population expansion over the last 125,000 years with the most intense increase in the last 35,000–25,000 years. Nuclear markers also revealed some increase in the last 50,000 years, but less marked than the mitogenomes did. This difference may be due to the smaller sample size of nuclear genes compared to that of mitogenomes. Nuclear markers also showed a more recent (in the last 19,000 years) population increase.

There is clear evidence of a strong glaciation in Colombia which began around 116,000 ya [121,122]. In the Tolima Department, it was detected a moraine dated to 100,000 ya, supporting an origin derived from this last glaciation that began around 116,000 ya [123]. Additionally, a very cold period, ca. 70,000 ya, produced very large glaciers (Nevados) in Colombia [124]. However, curiously, during this last phase of the Pleistocene and Holocene, *T. pinchaque* did not show evidence of population decrease as other Neotropical mammals did [125,126,127]. Henceforth, the strong cold periods of the fourth Pleistocene glaciation didn’t seem to affect the demography of *T. pinchaque.* Nevertheless, the major peaks of population expansion agree well with the glacier retreats in Colombia from 35,000 to 25,000 ya [128]. The nuclear markers detected population expansions around 19,000, and 11,000 ya. From 14,000 to 11,000 ya, precipitation and temperature increased, and glacial retreat again occurred. This allowed the formation of a lake (Tauca Lake; 43,000 km^2^) in the southern Bolivian Altiplano, which lasted until 11,000 ya [129]. This climatic change as detected in the Colombian Cundinamarca Department (named as Guantia period) where there was a rise of lake levels in the Savanah of Bogotá coupled with expansion of oak forests [128]. Some of the *T. pinchaque* population expansions correlated well with this period. However, then, an extremely cold period (11,000–10,000 ya) occurred. This period (named “El Abra” in the northern Andes) [130] had a crucial effect on some large mammals, such as Gomphoteriidae (proboscideans), giant sloths, large notoungulates, and the famous *Smilodon*, causing their extinction. Despite this, this extreme cold period did not seem to affect the demography evolution of *T. pinchaque*. Additionally, there is also evidence of population expansion during the Holocene for this species (around 3000 ya). The Holocene, specifically the Platense period, from 10,000 ya until the 16th century, was characterized by a biozone of *Lagostomus maximus* (plains viscacha). During this period the temperatures increased to a maximum at ca. 6000 ya. In the Savanah of Bogotá, the lake levels have increased in the last 9500–7000 years, and the Andean forests have expanded. According to some authors [131], around 6300 ya, there was a significant increase in temperature, especially in southern South America as well as in the Central Andes, evidenced from ^18^O levels in Huascaran snow. At ca. 5000–4500 ya, the level of the Titicaca Lake increased and around the 10th century BC, the temperature also increased [129]. But a dry interval Holocene period was detected as well. This dry period around 5000 ya was detected in the Amazon, Caquetá and lower Magdalena River basins as well as in the Andean lagoons of Colombia and Peru [132,133,134]. Curiously, again, *T. pinchaque* seems to have not been affected by these Holocene dry periods. Nonetheless, with retreats of the glacial and with the augment of humidity, *T. pinchaque* experienced significant population expansions detected both by mitochondrial and nuclear genes.

Future studies should analyze more specimens of *T. pinchaque*, and molecular markers, in the most northern areas of the Colombian Central Cordillera where the species lives as well as in the Colombian Eastern Cordillera and northern Peru for detecting possible different gene pools (MUs or ESUs) [135]. On the other hand, this study, including nuclear DNA sequences, seems to demonstrate the inexistence of *T. kabomani* as a full species. Nevertheless, future analyses with DNA from bones of fossil tapirs could help clarify the real number of taxa or species of tapirs in the Neotropics during the Pliocene and Pleistocene.

## Figures and Tables

**Table 1:** Geographical origins of the 44 Andean or mountain tapirs (*T. pinchaque*) sampled for complete mitogenomes. The specimens sampled for nuclear genes (*RAG 1-2*, *IRBP*, and *BRCA1*) were sub-sets of these 44 specimens.

*T. pinchaque*	Country	Department or Province	Locality	Number of Specimens
	Colombia	Caldas (3)	Los Nevados National Park	3
		Risaralda (5)	El Bosque/Los Nevados National Park	5
		Tolima (9)	Hereje River	1
			Gaitana	3
			Saldaña River	1
			Marquetalia	1
			Peñas Blancas	1
			La Planada	1
			La Azulena	1
		Huila (2)	Puracé National Park	1
			Nevado del Huila National Park	1
	Ecuador	Sucumbios (1)	La Bonita	1
		Napo (20)	Chaco/Sarañán	3
			Cosanga	2
			Cuyuja	6
			Cayambe-Coca National Park	5
			Papallacta	4
		Morona-Santiago (3)	Sangay National Park	3
		Loja (1)	Podocarpus NP	1

**Table 2:** Different molecular and chromosomal studies where the temporal splits between different tapir species were estimated. mya = millions of years ago.

Study	Marker	Split Between *T. bairdii/T. terrestris–T. pinchaque*	Split Between *T. terrestris* and *T. pinchaque*
[23]	mt *COII*	20–18 mya	2.7–2.5 mya
[24]	mt *12S rRNA*	16.5–15 mya	1.6–1.5 mya
[82]	Chromosomal rearrangements	19–18 mya	4.8–3.9 mya
[25]	mt *Cyt-b*	10–9 mya	3.8–1.6 mya
[4]	mt *Cyt-b*	7.5–3.1 mya	<0.13 mya
[8]	15 mt genes	8.1 mya	3.7 mya
Current work	Mitogenomes and three nuclear genes	6.2 mya	1.9 mya

## Data Availability

The data sets generated and analyzed during the current study are available from the corresponding author on reasonable request at the e-mails, mruizgar@yahoo.es, and mruiz@javeriana.edu.co. The GenBank accession numbers of some tapirs analyzed are OR685379-OR685429.
